# Dynamic magnetic field alignment and polarized emission of semiconductor nanoplatelets in a liquid crystal polymer

**DOI:** 10.1038/s41467-022-30200-2

**Published:** 2022-05-06

**Authors:** Dahin Kim, Dennis Ndaya, Reuben Bosire, Francis K. Masese, Weixingyue Li, Sarah M. Thompson, Cherie R. Kagan, Christopher B. Murray, Rajeswari M. Kasi, Chinedum O. Osuji

**Affiliations:** 1 Department of Chemical and Biomolecular Engineering, University of Pennsylvania, Philadelphia, PA 19104 USA; 2 Department of Chemistry, University of Connecticut, Storrs, CT 06269 USA; 3 Polymer Program, Institute of Materials Science, University of Connecticut, Storrs, CT 06269 USA; 4 Department of Chemistry, University of Pennsylvania, Philadelphia, PA 19104 USA; 5 Department of Electrical and Systems Engineering, University of Pennsylvania, Philadelphia, PA 19104 USA; 6 Department of Materials Science and Engineering, University of Pennsylvania, Philadelphia, PA 19104 USA

**Keywords:** Organic-inorganic nanostructures, Liquid crystals, Polymers, Quantum dots

## Abstract

Reconfigurable arrays of 2D nanomaterials are essential for the realization of switchable and intelligent material systems. Using liquid crystals (LCs) as a medium represents a promising approach, in principle, to enable such control. In practice, however, this approach is hampered by the difficulty of achieving stable dispersions of nanomaterials. Here, we report on good dispersions of pristine CdSe nanoplatelets (NPLs) in LCs, and reversible, rapid control of their alignment and associated anisotropic photoluminescence, using a magnetic field. We reveal that dispersion stability is greatly enhanced using polymeric, rather than small molecule, LCs and is considerably greater in the smectic phases of the resulting systems relative to the nematic phases. Aligned composites exhibit highly polarized emission that is readily manipulated by field-realignment. Such dynamic alignment of optically-active 2D nanomaterials may enable the development of programmable materials for photonic applications and the methodology can guide designs for anisotropic nanomaterial composites for a broad set of related nanomaterials.

## Abstract (web summary)

Liquid crystals (LC) are promising materials for the fabrication of reconfigurable arrays of 2D nanomaterials but it remains difficult to achieve stable dispersions of nanomaterials. Here, the authors report on good dispersions of pristine CdSe nanoplatelets (NPLs) in LCs, and reversible, rapid control of their alignment and associated anisotropic photoluminescence using a magnetic field.

## Introduction

Composite materials constituted by nanoparticles (NPs) dispersed in liquid crystals (LCs) have attracted interest as a new type of stimuli-responsive functional material^1–3^. This interest is driven by the potential to use the stimuli-responsive director field of LCs to control the positional and orientational order of NPs with useful properties^4–6^. Positional order can be programmed by carefully exploiting interparticle interactions driven by director field distortion, and the tendency of NPs to localize at topological defects in the LC^7–9^. Orientational order likewise is imposed by the director field coupling to the structural anisotropy of the NP^10–16^. The anchoring condition of LC mesogens at the surface of NPs plays a critical role in dictating the resulting interactions and director field coupling that lead to positional and orientational control, respectively. In practice, however, the realization of such stimuli-responsive nanocomposites is hampered greatly by the difficulty of producing stable dispersions of NPs in LCs above vanishingly small volume fractions. Dispersion stability is diminished by energetic penalties associated with any director field distortion due to the NP, and the excess free energy that arises due to chemical incompatibility of the NP surface with the LC medium. If there is director field distortion, the LC host expels NPs to minimize the total elastic energy^17–19^, and unfavorable surface interactions result in phase separation of NPs unless the interactions are offset entropically^20^.

Minimizing or eliminating director field distortion requires that the anchoring condition is matched to the symmetry of the NP. Homeotropic anchoring (i.e. perpendicular to the surface) of rod-like mesogens at any curved surface mandates the formation of a topological defect, and splay deformation of the mesophase. Planar anchoring (i.e. parallel to the surface) on a sphere, or along the circumference of a cylinder or rod, likewise induces distortion of the LC. Homeotropic and planar anchoring at flat surfaces, and planar anchoring parallel to the long axis of a cylindrical or rod-like NP inclusion, can be accomplished with little or no director field distortion. Minimizing the energetic penalty associated with the chemical incompatibility of the LC and the NP can be accomplished by modifying the NP surface chemistry using appropriate ligands. The director distortion and surface energy contributions to dispersion stability are coupled as the mesogen anchoring, particularly at smooth surfaces, is strongly influenced if not completely dictated by NP surface chemistry. Surface conditions to satisfy both together are often restricted as the surface anchoring and elastic energetic costs are competing each other. Prior reports have demonstrated spatial localization of spherical NPs in highly dilute dispersion in nematic fluids^21–25^ and orientational order in dispersions of anisotropic nanomaterials, particularly nanorods^10–16^. This limited success to date in the development of functional NP-LC materials is properly viewed in the context of the challenging dispersion problem discussed above.

Here, our focus is centered on controlling orientational order of two-dimensional (2D) nanomaterials, and specifically, CdSe nanoplatelets (NPLs). CdSe NPLs present unique anisotropic optoelectronic features due to strong quantum confinement along their thickness direction, and their atomically uniform thickness enables narrow photoluminescence linewidth compared to other CdSe nanomaterials^26,27^. The development of CdSe NPLs assemblies with controlled orientation paves the way to harnessing their orientation-dependent optical properties in useful ways; their properties of interest include linearly polarized emission in the “edge-on” orientation and enhanced light extraction in the “face-on” configuration^28–30^.

Native ligands with long alkyl tails (i.e. oleic acid) of CdSe NPLs can provide a strong perpendicular boundary condition at the flat NPL-LC interface and the anchoring condition could minimize director field distortion by spontaneously aligning NPLs’ normal parallel to the director^31^. However, in spite of the relatively weaker director distortion at flat surfaces, there are few examples of 2D NP-LC composites including high contents of particles, and no reports on CdSe NPLs^6,10,32^. 2D nanomaterials present additional challenges regarding dispersion in LCs as strong interparticle interactions (e.g. van der Waals, hydrophobic, and depletion attractions) between extended flat faces easily lead to aggregation^33^. Destabilization factors in a 2D NP-LC system needs to be further studied and the processing pathway should be designed toward minimizing not only energetic penalties arising from NP–LC interaction but also interparticle attraction.

In this study, we demonstrate a simple yet effective approach to make long-range orientationally ordered dispersions of CdSe NPLs and CdSe/ZnS core/shell NPLs in LCs, and magnetic field-tunable polarized emission characteristics. Stable dispersions of NPLs were achieved using only the native oleic acid ligands present during NPL synthesis as the compatibilizing agent for an appropriate LC medium. The LC medium was a blend of side-chain liquid-crystalline polymers and labile small molecule mesogens; the labile mesogens are added to improve the kinetics of the magnetic field response and enable orientation switching at ambient temperature^34^. The blend system maintains colloidal stability of the NPLs from dilute solutions through solvent removal to solid films of field-aligned smectic nanocomposites. Magnetic field application leads to alignment of the NPLs with their surface normal parallel to the field. This alignment results from the alignment of the LC director parallel to the field and the homeotropic anchoring of the mesogens at the oleic acid functionalized NPL surface. Highly ordered states with orientation distribution coefficients, <P_2_^NPL^ > ~ 0.8 to 0.9, can be obtained with field strengths between 0.3 and 5.8 T, depending on temperature, and the alignment can be reversibly changed by altering the direction of the field. Consequently, aligned CdSe/ZnS NPLs exhibit linearly polarized emission perpendicular to the LC director with a degree of polarization of 0.54, and the ensemble polarization is also switchable with response to fields. The strong and tunable polarized emission observed over macroscopic length scales suggests potential applications as functional materials for photonic applications. The approach developed here can be leveraged in other NP–LC nanocomposites to enable orientational control and the development of materials with well-defined anisotropic functional properties.

## Results and discussion

### Stable dispersion of CdSe NPLs in LCs

**Fig. 1:**
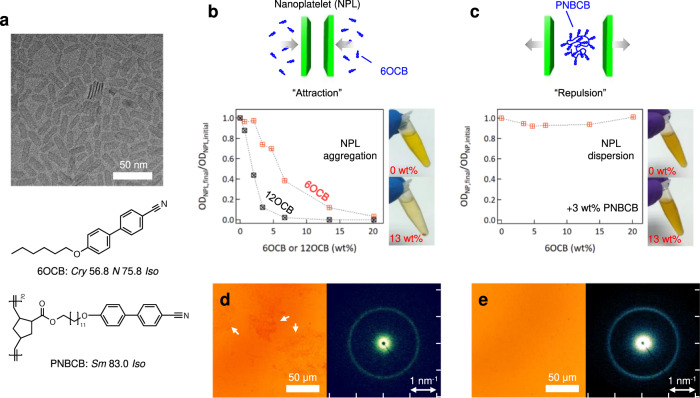
Dispersion stability of colloidal CdSe nanoplatelets (NPLs) in solution in the presence of liquid crystals (LCs). **a** TEM image of CdSe NPLs and molecular structures of 6OCB and PNBCB and their phase transition temperatures. **b** Stability of NPLs in chloroform with the addition of 6OCB or 12OCB, which is evaluated by the decrease in optical density (OD) of supernatants after gentle centrifugation of the solutions. Photographs of NPL solutions (top) without 6OCB and (bottom) with 13 wt.% 6OCB. **c** Stability of NPLs in PNBCB/6OCB blend solutions as a function of the 6OCB concentration with constant 3wt.% of PNBCB, and photographs of NPL/PNBCB solutions (top) without 6OCB and (bottom) with 13 wt.% 6OCB in the presence of 3wt.% PNBCB. **d** Optical microscopy (OM) image of an isotropic NPL/6OCB composite and the small-angle X-ray scattering (SAXS) result. **e** OM image of an isotropic NPL/PNBCB/6OCB composite and the SAXS data.

We synthesized CdSe NPLs according to a previously reported procedure with some modifications^35^. The TEM image in Fig. 1a reveals the uniform shape of the synthesized CdSe NPLs with average lateral dimensions of 24 × 9 nm. The atomically flat surface and quantum confinement along the NPL thickness direction result in ultra-narrow absorption and emission peaks corresponding to the CdSe thickness of 1.4 nm (Supplementary Fig. 1). The LC host for the NPLs is a blend of free mesogens, 4-cyano-4′-n-hexyloxybiphenyl (6OCB), and liquid-crystalline polymers constituted by a norbornene backbone functionalized by cyanobiphenyl mesogens with a 12-methylene spacer (PNBCB) (Fig. 1a and Supplementary Fig. 2). The phase behavior and birefringent textures of 6OCB and PNBCB as characterized by differential scanning calorimetry (DSC) and polarized optical microscopy (POM) are shown in Supplementary Figs. 3, 4. To make the NPL-LC composite, a mixture of NPLs and LCs in solution was prepared and the solvent was removed by evaporation above the clearing temperature of the LC medium, T_NI_. The resulting isotropic composite was cooled to produce a LC mesophase. The miscibility and colloidal stability of NPLs with LC hosts were examined at each step in the above process, i.e. in solvent suspension, in the isotropic composite (T > T_NI_), and in the LC mesophase (T < T_NI_).

Stability was monitored using the decrease in optical density resulting from a severe NPL aggregation and ensuing sedimentation (Supplementary Fig. 5) as a function of mesogen or polymer concentration in solution with the NPLs. As seen in Fig. 1b, the presence of 6OCB and 12OCB mesogens leads to a loss of stability, with higher mesogen concentrations yielding greater aggregation. 12OCB causes a larger loss of stability than 6OCB at equivalent mass concentration. By contrast, strikingly, the presence of the polymer PNBCB at 3 wt.% results in negligible loss of stability on the addition of 6OCB, over the same range of concentrations (0–20 wt.%) considered with 6OCB and 12OCB only (Fig. 1c). The loss of stability in the presence of 6OCB or 12OCB alone is presumably due to depletion-induced attraction of the NPLs. The strength of depletion-induced attraction is proportional to the number density of depletants, which is consistent with the decrease in suspension stability on increasing mesogen concentration. Given its higher molar mass, 12OCB is present at a lower number density at equal mass concentration than 6OCB which suggests it should not destabilize the NPLs as much as 6OCB at the same concentration. However, the range of the induced attraction scales with the size of the depletant, and this may account for the observed difference. We note in passing that the aforementioned challenge associated with stabilizing plate-like vs. spherical particles is highlighted by the contrasting behavior of 2.7 nm CdSe (spherical) NPs, which remain stable in the presence of 6OCB and 12OCB (Supplementary Fig. 6).

The behavior observed with added LC polymer, despite the presence of 6OCB, indicates that the polymer has a strong stabilizing effect on the NPL dispersion. The impact of polymers on colloidal stability can be complex as it depends on the nature of the polymer-colloid surface interaction, and the polymer concentration and molar mass^36,37^. Recent experimental and theoretical results show that a polymer-induced depletion interaction in non-adsorbing systems has a short-range attractive well, but also a longer-range repulsive barrier^38,39^. At concentrations in the semi-dilute regime, the repulsive barrier can become sufficiently high to confer kinetic stability, with interparticle separations similar to the length scale of polymer concentration fluctuations. Conversely, a sequence of bridging-induced aggregation, steric stabilization, and depletion-induced aggregation on increasing polymer concentration is well-known for surface-adsorbing polymers. It is not yet clear whether the stability observed here is due primarily to steric stabilization, or to the formation of a depletion-induced repulsive barrier. However, studies conducted as a function of polymer concentration for two different molar masses suggest that the stability may be associated with a depletion-induced repulsive barrier (Supplementary Fig. 7).

The colloidal stability of NPL suspensions strongly affects the quality of the final solvent-free nanocomposites produced after solvent evaporation. Nanocomposite films were observed by bright-field optical microscopy (OM) and small-angle X-ray scattering (SAXS) in their isotropic state (T > T_NI_). The NPL/6OCB nanocomposite shows clear signs of phase separation of NPLs within the film, with irregularly shaped agglomerates up to tens of microns in size clearly visible in OM (Fig. 1d). SAXS shows a well-defined peak at scattering vector *q* = 1.26 nm^−1^, indicating that the agglomerates are constituted by stacked platelets with a center-center distance of *d* = 2π/q = 5.0 nm. Accounting for the 1.4 nm thickness of the NPLs, the separation between the oleic-acid functionalized faces is therefore 3.6 nm. By contrast, NPL aggregates could not be discerned in OM images of isotropic-state NPL/PNBCB/6OCB nanocomposites (Fig. 1e). The difference in visible signs of aggregation in these two solvent-free films is consistent with the difference in stability seen in the precursor suspensions, but also highlights that the PNBCB confers stability throughout the solvent evaporation process. SAXS data from NPL/PNBCB/6OCB films show that CdSe NPLs exist as 5 nm periodicity stacks, but the stacks, or any aggregates of stacks, are too small to be resolved by OM.

DSC, SAXS and POM measurements (Supplementary Figs. 3, 8, 9, and 10) for the PNBCB/6OCB blend without NPLs indicate that the system forms a smectic mesophase over a wide temperature range (~0–72 °C), with a small nematic window (~72–78 °C), inferred from POM and SAXS, before clearing to the isotropic phase at higher temperatures. The smectic has a layer spacing of 4.8 nm. Application of a magnetic field to the blend in the smectic phase at room temperature results in strong alignment of the LC director parallel to the field. (Supplementary Figs. 8, 11). Samples with NPLs show near-identical phase behavior and clearing temperatures as the blend without NPLs (Supplementary Fig. 3).

**Fig. 2:**
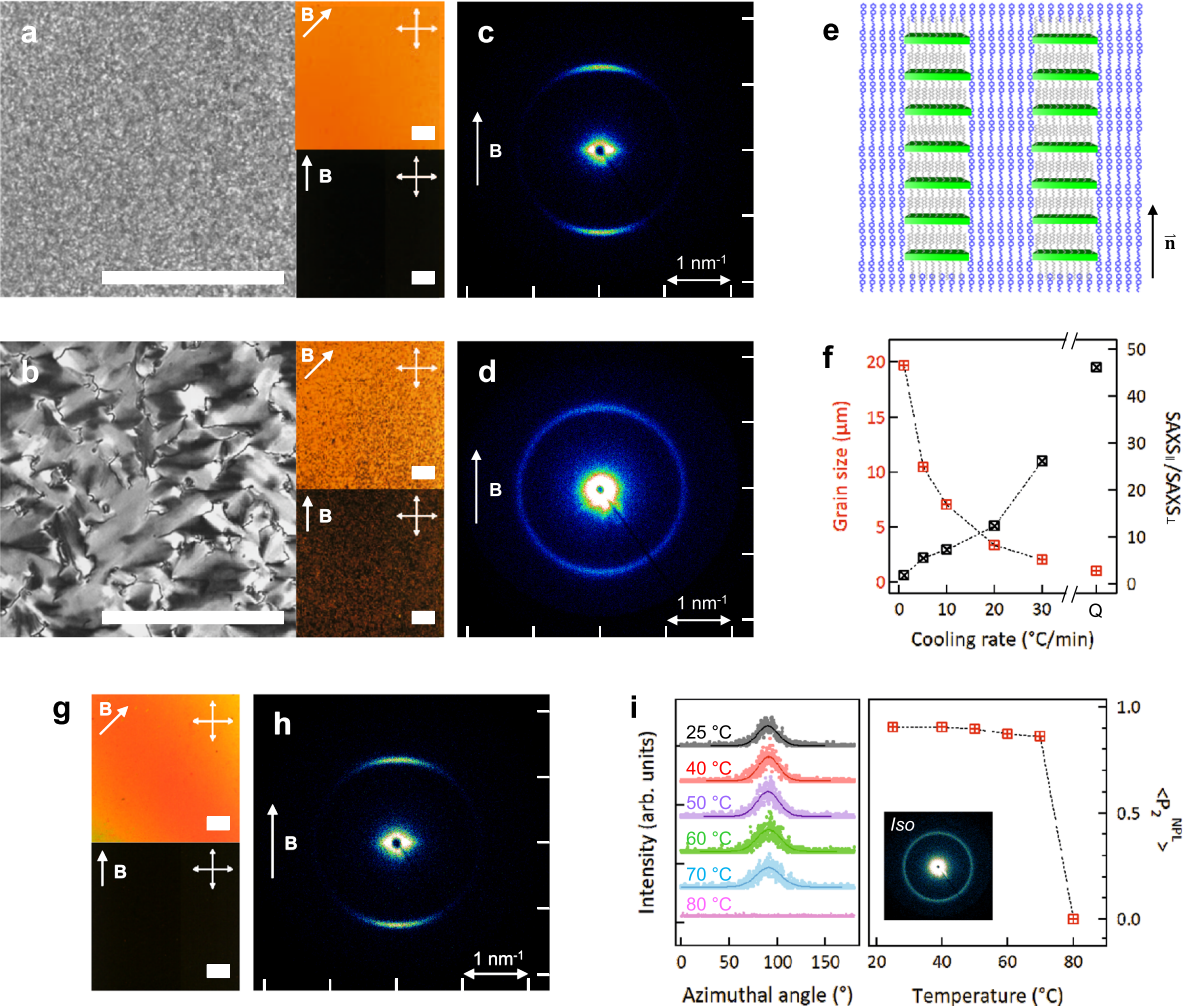
Dispersion stability of CdSe NPLs in the smectic phase of PNBCB/6OCB LC blends. **a** Polarized optical microscopy (POM) images of a NPL/PNBCB/6OCB composite film which was quenched (>30 °C/min) from the isotropic phase to room temperature; the left large image was taken without magnetic fields, and after a field of 5.8 T was applied, the right two images were obtained upon the sample rotation. The direction of the magnetic field (**B**) is indicated by the arrow. **b** POM images of a NPL/PNBCB/6OCB composite film which was slowly cooled at 1 °C/min from the isotropic phase to room temperature; the left large image was taken without fields and the right two images were bright and dark states after the field-induced in-plane alignment. **c**, **d** SAXS patterns of NPL/PNBCB/6OCB composites (**c**) quenched and (**d**) slowly cooled at 1 °C/min under vertical magnetic fields, respectively. **e** Schematic illustration showing NPL alignment in a PNBCB/6OCB matrix. The $\overrightarrow{{{{{{\bf{n}}}}}}}$ denotes the director of LCs. **f** Average grain size of smectic phase NPL/PNBCB/6OCB composites as a function of cooling rate, and contrast ratio of the scattered intensities at *q* = 1.26 nm^−1^ parallel (SAXS_||_) and perpendicular (SAXS_⊥_) to magnetic fields. The cooling rate, *Q*, represents a quenched sample. **g** Bright and dark states under crossed polarizers for a NPL/PNBCB/6OCB composite which was held at 70 °C for 30 min. **h** SAXS pattern of the sample corresponding to (**g**). **i** (left) Azimuthal intensity profiles fitted with Gaussian function for aligned NPL/PNBCB/6OCB composites depending on temperature. (right) Order parameter <*P*_2_^NPL^ > of NPLs in the composites as a function of temperature. Inset shows the isotropic SAXS pattern obtained at 80 °C. All scale bars represent 100 μm.

Nanocomposites of NPL in PNBCB/6OCB were cooled from the isotropic state into the smectic mesophase at room temperature. Microscopy and SAXS data from the resulting films show that the system response is sensitive to the rate of cooling. Samples that were quenched (cooling rate > 30 °C/min) to room temperature showed a fine texture in POM (Fig. 2a, left), whereas slowly cooled (1 °C/min) samples showed large grains (Fig. 2b, left). The smectic phase composites were then subjected to a 5.8 T magnetic field for LC alignment. Phase separation was observed in the dark and bright-field images of slowly cooled samples after the alignment (Fig. 2b, right), whereas no such large aggregates were visible in the quenched systems (Fig. 2a, right). Correspondingly, we observe azimuthally uniform SAXS intensity from the CdSe NPL stacking peak at *q* = 1.26 nm^−1^ for slowly cooled samples (Fig. 2d), but highly anisotropic scattering at the same scattering vector for quenched samples (Fig. 2c). Taken together, the data indicate that uniformly dispersed small NPL stacks are aligned along the field direction for quenched samples, as schematically illustrated (Fig. 2e), whereas largely aggregated NPLs with no preferred orientation result from slow cooling^40,41^. The native oleic acid ligands induce homeotropic anchoring of LC host mesogens at the NPL surface. The low q streak-scattering along the equatorial line for quenched samples is consistent with the presence of colloidal scale objects with sharp interfaces, as provided by elongated CdSe NPL stacks aligned parallel to the applied magnetic field. The smectic layer reflection at 1.3 nm^−1^ is obscured by the NPL stacking peak.

The importance of the cooling rate is highlighted by examining the LC grain size and anisotropy of X-ray scattering as a function of cooling rate (Fig. 2f). Rapid cooling produced small grains within which the NPL stacks are uniformly distributed and can be strongly aligned under external fields, and there is a continuous increase in grain size and a decrease in the degree of alignment with decreasing cooling rate. The dependence of grain size on cooling rate is also observed in the PNBCB/6OCB host without NPLs (Supplementary Fig. 12). The transition temperatures and enthalpies are not affected by the cooling rate for the LC host and nanocomposite (Supplementary Fig. 13).

To clarify the thermal effects, we examined the influence of isothermal annealing on the phase separation of NPLs. Samples held for 30 min at 70 °C show no evidence of phase separation. POM shows uniform transmission and extinction of light as a function of crossed-polarizer orientation with respect to the field, with no visible aggregates (Fig. 2g). Correspondingly, the SAXS shows a strong azimuthal intensity concentration along the meridional line, consistent with the preservation of NPL stacks aligned parallel to the field (Fig. 2h). Similar results were obtained for temperatures lower than 70 °C, as inferred by the retention of anisotropic scattering whereas NPL alignment is gradually lost when kept at 74 °C (Supplementary Fig. 14). At the isotropic phase (80 °C), the alignment is completely lost, as shown by temperature-dependent azimuthal intensity profiles and orientational order parameters in Fig. 2i, extracted from temperature-dependent SAXS (Supplementary Fig. 14). It is therefore apparent that the NPL stacks lose stability in the nematic window, and the alignment sensitivity to cooling rate is related to the residence time in the nematic during processing, with slowly cooled samples undergoing a phase separation process in the nematic state. We are not aware of similar phenomena in other colloid-LC systems and a detailed microscopic picture regarding the differential stability in the nematic vs. smectic phases is not available. We surmise, however, that the symmetry of the layered smectic phase is more compatible with the 2D structure of the NPLs, and that the near coincidence of NPL stacking distance (5 nm) with the smectic layer spacing (4.8 nm) are contributing factors. Additionally, the restricted mobility across the smectic layers may play a role for any kinetic considerations regarding stacking and potential phase separation. The LC phase governs the dispersion stability and the orientational order of NPL components. However, the correlation lengths of NPL stacks are not significantly affected by phase and temperature but only determined by the type of LC medium (Supplementary Fig. 15). NPLs have a larger correlation length in the LC blend system than in 6OCB. This reflects the tendency to form random clusters in 6OCB where the NPLs are not stable dispersed, vs. well-assembled stacks in the LC blend system where they are stable.

We also demonstrated the alignment of NPLs in a cross-linkable system, using RM257 instead of 6OCB as free mesogen (Supplementary Fig. 16). After magnetic alignment and UV curing, the resulting composite polymer preserves the aligned morphology in the absence of external field. In TEM measurement, they exhibit alignment of NPL stacks along the LC director, providing visual evidence for the stable NPL alignment in the LC blend.

### Rapid and reversible magnetic alignment of CdSe NPLs in LCs

**Fig. 3:**
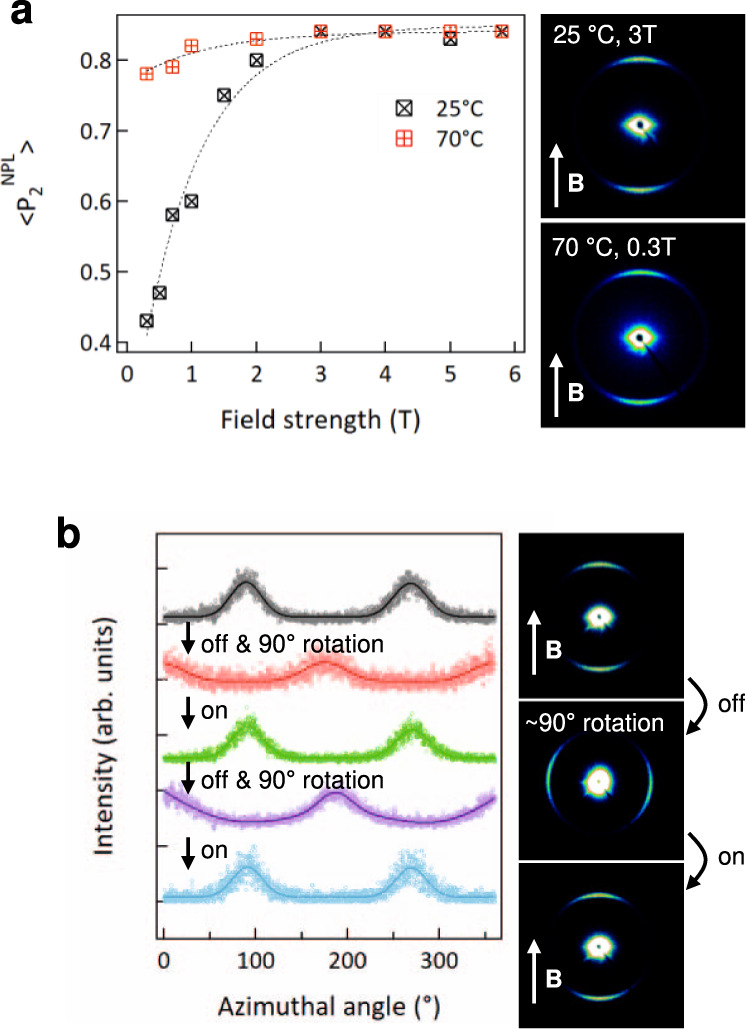
Field strength dependence of NPL alignment and reversibly tunable NPL orientation by fields. **a** (left) Order parameter <*P*_2_^NPL^ > of NPLs in PNBCB/6OCB as a function of field strength at two different temperature conditions, 25 and 70 °C. (right) SAXS patterns obtained at 25 °C and 3 T and at 70 °C and 0.3 T, respectively. The direction of the magnetic field (**B**) is indicated by the arrow. **b** (left) Change in an azimuthal intensity profile of a NPL/PNBCB/6OCB composite upon field on/off and sample rotation and (right) the corresponding SAXS patterns. Initially, the composite was aligned using a “vertical” field at 25 °C and 3 T. It was then rotated ~90° in the absence of the field (0 T) around the direction of X-ray incidence, such that the original magnetic field direction, and the resulting LC director field, were now “horizontal”. Upon applying the field again in the vertical direction, the NPL orientation fully reverted within 10 s as the LC director field realigned from horizontal to vertical. Additional experiment involving a 90° turn in the opposite direction demonstrates the reversible reconfiguration.

The field strength dependence of NPL alignment was examined at two different temperatures for quenched samples. As shown in Fig. 3a, the degree of alignment is sensitive to field strength and to temperature. The alignment saturates (<*P*_2_^NPL^ > ~0.84) at a characteristic field of ~3 T at 25 °C. By contrast, at 70 °C, the characteristic field required to align is about an order of magnitude smaller, ~0.3 T. The addition of the labile mesogens significantly reduces the viscosity of the LC blend and allows the low-intensity field alignment, although PNBCB lacks mobility because of high viscosity. The temperature dependence also highlights the kinetic aspect of alignment, as the viscosity of the LC host decreases with temperature. The ability to quickly change the orientation of the NPLs was considered by subjecting an aligned sample to a new field direction. A sample with alignment along the meridional line was rotated 90° such that the NPL stacking peak migrated to the equatorial line. Activation of the 3 T magnetic field at room temperature resulted in a rapid return of the scattering to the meridional line, as shown in Fig. 3b. The realignment was rapid—it occurred within 10 s, which is the smallest duration resolvable by the scattering experiment. The alignment was also reversible, as shown by the azimuthal intensity plots. The rapid alignment and realignment kinetics suggest that dynamic control of the NPL orientation can be feasibly enacted in these systems using modest magnetic fields accessible by simple permanent magnets at modest temperatures.

### Stable dispersion of highly emissive CdSe/ZnS NPLs in LCs

**Fig. 4:**
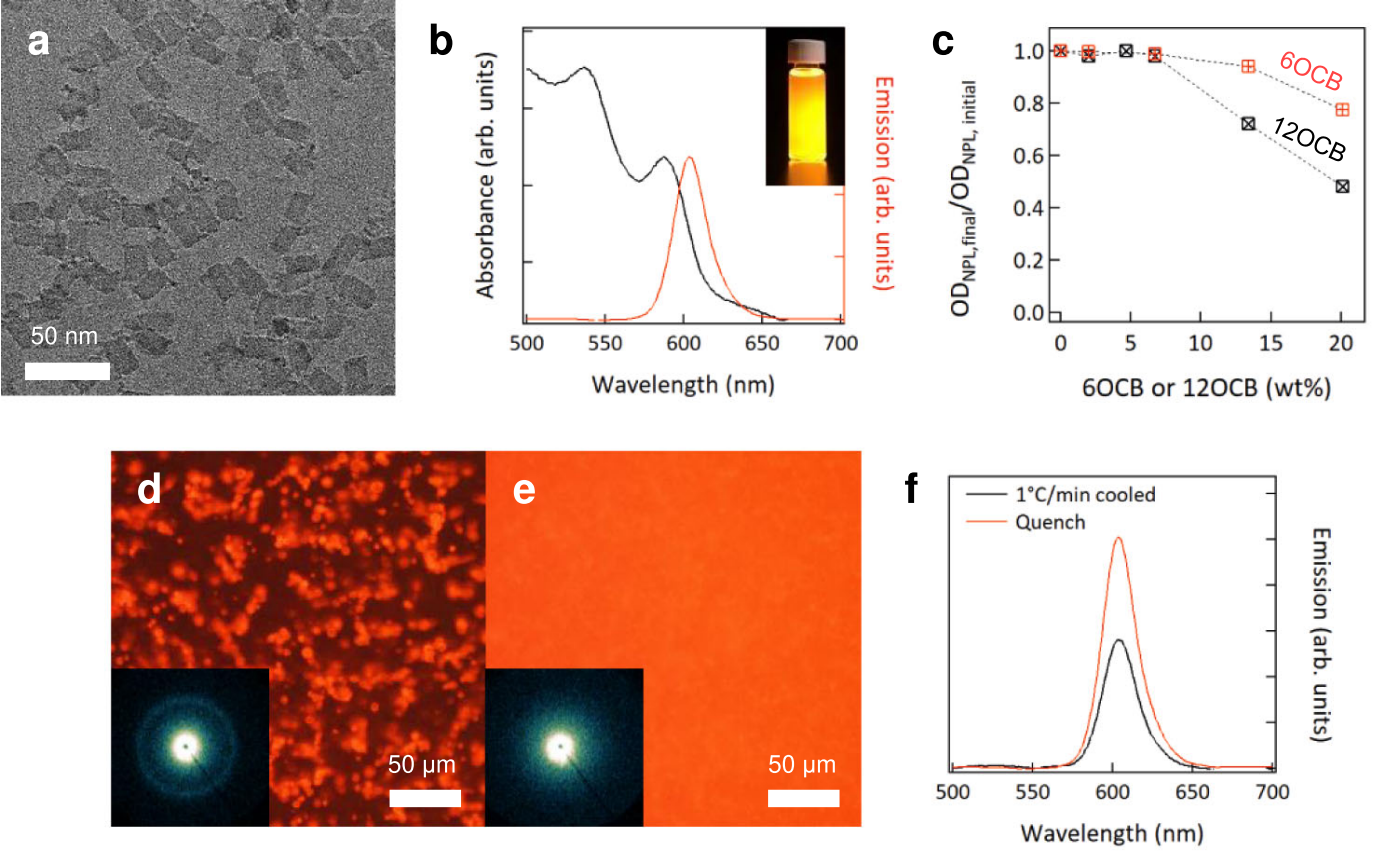
Dispersion stability of CdSe/ZnS core/shell NPLs in PNBCB/6OCB. **a** TEM image of CdSe/ZnS NPLs. **b** Absorption and emission spectra of CdSe/ZnS NPLs. Inset shows a photograph of the NPL solution emitting orange light under UV irradiation. **c** Stability of NPLs in chloroform as a function of the concentration of 6OCB and 12OCB. This is quantified by the decrease in OD of supernatants after gentle centrifugation of the solutions. **d**, **e** Fluorescence microscopy (FM) images of NPL/PNBCB/6OCB composite films obtained (**d**) by slow cooling at 1 °C/min and (**e**) rapidly quenching from the isotropic phase to room temperature, respectively. Insets show the corresponding SAXS patterns. **f** Photoluminescence intensity of a slowly cooled or quenched composite film, measured using a spectrometer with an integrating sphere.

Our results show that CdSe NPLs can be stably dispersed and aligned/realigned magnetically. However, the optical properties of CdSe NPLs, and specifically their fluorescence emission is compromised in the absence of a protective layer^42,43^. We therefore prepared ZnS passivated CdSe NPLs. The ZnS shell preserves the attractive emissive properties of the CdSe NPLs in a wide variety of dielectric environments including that of our PNBCB/6OCB host. TEM image of CdSe/ZnS NPLs (Fig. 4a) shows that the 2D NPL shape is maintained. The NPL photoluminescence spectrum is red-shifted from 513 to 605 nm (Fig. 4b), resulting in orange fluorescence under UV light as shown in the inset. This red shift occurs because of a reduction in the 1D confinement of the exciton. The SAXS pattern of drop-casted core/shell NPLs shows a peak at smaller scattering vector (*q* = 0.98 nm^−1^) than that of CdSe NPLs_,_ which implies that the face-to-face distance is increased from 5 nm to 6.4 nm due to the ZnS shell (Supplementary Fig. 17). The stability tests conducted for CdSe/ZnS NPLs show that core/shell NPLs have a better colloidal stability than CdSe NPLs but still exhibit considerable settling in solutions containing only 6OCB or 12OCB when PNBCB is absent (Fig. 4c and Supplementary Fig. 18).

The strong emission of CdSe/ZnS NPLs allows to visualize their dispersion in the PNBCB/6OCB matrix using fluorescence microscopy (FM) (Fig. 4d, e). Slowly cooled samples (1 °C/min) result in phase separation of NPLs and the resulting fluorescence is therefore heterogeneous (Fig. 4d). Overlaid POM and FM images suggest that NPLs aggregate at grain boundaries and other defects in the LC (Supplementary Fig. 19). SAXS exhibits an isotropic ring at *q* = 0.98 nm^−1^ corresponding to the 6.4 nm spacing of the NPL stacks. By contrast, quenched nanocomposite films display uniform orange emission without visible signs of aggregation (Fig. 4e). Isothermal annealing of the quenched films at the smectic does not cause any change on the optical features whereas non-uniform fluorescence emerges when kept at the nematic (Supplementary Fig. 20). Interestingly, SAXS from quenched samples does not exhibit a distinct scattering ring from stacked NPLs but instead shows only diffuse scattering (Inset of Fig. 4e). This suggests that NPL stacks do not form and the NPLs are instead singly dispersed, which is also supported by TEM observation (Supplementary Fig. 21). Dispersion as discrete objects has a beneficial effect on the fluorescence intensity as it can exclude the possibility of ultrafast energy transfer and ensuing nonradiative recombination occurring in closely-stacked NPLs. The brighter emission observed from quenched films relative to slowly cooled films as seen in Fig. 4f is consistent with this picture.

### Dynamic control of polarized emission of macroscopic NPL-LC composites

**Fig. 5:**
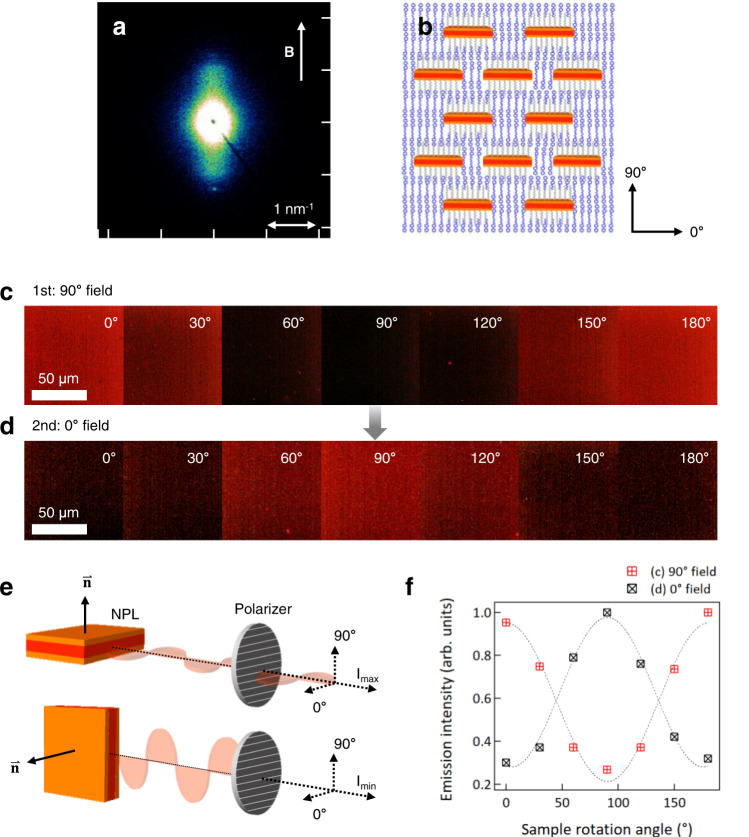
Dynamic alignment of CdSe/ZnS core/shell NPLs in PNBCB/6OCB and the polarized emission characteristics. **a** SAXS pattern of NPL/PNBCB/6OCB composites under field of 5.8 T at 50 °C. **b** Schematic presentation illustrating CdSe/ZnS NPL dispersion and alignment in a smectic phase PNBCB/6OCB matrix. **c**, **d** FM images of a field-aligned NPL/PNBCB/6OCB film as a function of the sample rotation angle, **c** after alignment under 90° field and (**d**) realignment under 0° field. The polarizer axis in the emission pathway is always along 0°. **e** Schematic illustration for the case of (**c**), showing the origin of the bright and dark state according to the orientation of NPLs relative to the $\overrightarrow{{{{{{\bf{n}}}}}}}$ axis. **f** Normalized emission intensity of the aligned NPL/PNBCB/6OCB composite film as a function of the sample rotation angle, determined from the FM images shown in (**c**) and (**d**).

SAXS from field-aligned samples is shown in Fig. 5a. The alignment of the smectic layers is evident from the spot-like reflection at *q* = 1.3 nm^−1^ while diffuse scattering along the meridional direction indicates the alignment of single dispersed CdSe/ZnS NPLs with their surface normal parallel to the LC director, as shown in Fig. 5b^41,44–46^. The anisotropy of the diffuse scattering is shown in Supplementary Fig. 22. These results demonstrate that NPLs are well-dispersed as single objects and well-aligned, with an orientational order parameter <*P*_2_^NPL^ > ~0.84, as estimated from the azimuthal intensity scan at *q* = 0.98 nm^−1^ (Fig. 5b and Supplementary Fig. 22).

Aligned samples for emission polarization measurements were fabricated by sandwiching the quenched nanocomposite films between glass substrates and applying in-plane magnetic fields. Emission polarization was characterized using an optical microscope equipped with an unpolarized excitation source and a polarizer in the emission path on the way to a CCD camera. The angle-dependent fluorescence intensity is determined by rotating the sample stage relative to other components. Because NPLs are field-oriented with their edges perpendicular to the director (Fig. 5b), the light emitted by the films is linearly polarized with no polarization degeneracy. Figure 5c shows the angle-dependent fluorescence intensity, where the angle is defined relative to the polarizer (polarizer at 0° transmits light with electric field oscillations along this direction), with reference directions indicated in Fig. 5b. NPL surface normal alignment at 90° (field along 90°) results in the brightest emission at 0° and 180° whereas the emission intensity is at its minimum at 90°. A magnetic field applied orthogonal to the initial alignment direction results in NPL realignment, and the corresponding FM images (Fig. 5d) are phase-shifted by 90° relative to the initial response in Fig. 5c. Figure 5e summarizes the angular relationship among the field direction (**B**), LC director ($\overrightarrow{{{{{{\bf{n}}}}}}}$), NPL and polarizer. The brightest emission is obtained when the polarizer direction is in the plane of the NPLs, or conversely, when the NPL surface normal is orthogonal to the polarizer axis. The angular dependence of emission intensity follows the canonical cos^2^ dependence for both configurations (Fig. 5f). The emission polarization, defined as (I_||_−I_⊥_)/(I_||_+I_⊥_), where I_||_ and I_⊥_ are emission intensity parallel (perpendicular to $\overrightarrow{{{{{{\bf{n}}}}}}}$) and perpendicular (parallel to $\overrightarrow{{{{{{\bf{n}}}}}}}$) to the NPL alignment direction, respectively, is calculated as 0.54. Notably, this degree of polarization is higher than seen previously for mechanically or electrically aligned systems in which the NPL long axis direction is controlled. Such alignment produced a rotationally degenerate state, with a random distribution of faces and edges normal to the applied force direction^29,47^. By contrast, here, due to the LC anchoring and positive magnetic susceptibility, the field specifies the orientation of the NPL surface normal, which produces a non-degenerate state. The higher polarization emission is consistent with this.

In summary, in this study, we describe magnetic alignment of 2D NPLs stably dispersed in a LC polymer, and the resulting anisotropic optical properties of such assemblies. Stable dispersions in a smectic mesophase were realized by preventing aggregation of NPLs in solution, and by avoiding LC director field distortion through the use of homeotropic anchoring conditions. The stability in the nematic state was limited, however, which suggests that geometric compatibility of the sheet-like nanomaterials with the layered LC structure, and/or kinetic effects associated with reduced mobility in the smectic mesophase may play a role. Samples aligned rapidly in the smectic phase at ambient temperature in the presence of a magnetic field, resulting in strong alignment of the LC director and the NPL surface normal parallel to the field. The aligned NPLs display anisotropic emission, with a high degree of polarization. Field-induced realignment of the system was advanced readily, resulting in rapid change of the angular emission characteristics. Field control of the surface normal of the NPLs yields a population of particles with a well-specified orientation. This contrasts with the degeneracy seen to date in related materials aligned by shear flow or electric fields. This has important implications for sharpening the angular dependence of emission, and enables the realization of polymer-embedded NPLs with all “face-on” orientation. Such materials are highly sought after for active layers for LCD applications and other smart optoelectronic devices, including polarized photoconversion devices. We anticipate that the methodology developed here may be extended to enable the stable incorporation of diverse nanomaterials into LC polymers and to realize useful functional properties therefrom.

## Methods

### Chemicals

5-Norbornene-2-carboxylic acid (mixture of endo and exo, 98%), 4-dimethylaminopyridine (DMAP, 99%) and anhydrous pyridine (99.8 %) are purchased from Sigma-Aldrich. Dry Dichloromethane (DCM, 99.8%), oxalyl chloride (98%), and anhydrous dimethylformamide (DMF) are obtained from Acros Organics. 12-bromo-1-dodecanol (98%) and Cyano-4′-hydroxybiphenyl (98%) are obtained from TCI America. Grubb’s catalyst third generation are purchased from Sigma-Aldrich and used without further purification. 4′-cyano-4-hexyloxybiphenyl (6OCB, 96%) was purchased from Sigma-Aldrich and 4′-cyano-4-dodecyloxybiphenyl (12OCB, ≥98%) was purchased from TCI America. Cadmium nitrate tetrahydrate (Cd(NO_3_)_2_·4H_2_O, 99%), myristic acid (Myr, 99%), selenium (Se), zinc acetate (Zn(Ac)_2_, 99.99%), cadmium acetate (Cd(Ac)_2_, 99.995%), oleic acid (OA, 90%), 1-octanethiol (≥ 98.5%), oleylamine (OLA, 70%), and 1-octadecene (ODE, 90%) were purchased from Sigma-Aldrich.

### Synthesis of norbornene functionalized *n*-alkyloxy cyanobiphenyl, NBCB monomer

NBCB monomer was synthesized using modified literature procedures^48,49^. A round bottom flask equipped with a magnetic stir bar, was charged with 4-cyano-4′-hydroxybiphenyl (8.0 g, 41 mmol), K_2_CO_3_ (8.5 g, 62 mmol), 12-bromo-1-dodecanol (13.0 g, 49 mmol) and 100 mL of DMF. The flask was sealed, purged with nitrogen for 10 min and allowed to stir for 48 h. The reaction mixture was then cooled down, and intermediary product (CB12-OH) obtained after recrystallization using ethanol.

To a single neck flask charged with 5-norbornene-2-carboxylic acid (86/14 = endo/exo) (5.0 g, 36 mmol) and 25 mL of DCM under nitrogen atmosphere, excess oxalyl chloride (9.2 mL, 109 mmol) was injected slowly. Two drops of DMF were added and reaction allowed to stir for 6 h at room temperature. After removal of excess oxalyl chloride, the obtained norbornene chloride was diluted with 10 mL of DCM and added dropwise to a solution of CB12-OH (7.0 g, 18 mmol, 40 mL of DCM). Then a catalytic amount of DMAP in 1 mL of pyridine was added and reaction mixture allowed to stir under nitrogen atmosphere overnight. The final product, NBCB monomer was obtained after purification using column chromatography (silica gel as stationary phase, and hexane/ethyl acetate as the mobile phase) and recrystallization using ethanol.

### Synthesis of PNBCB homopolymers

A representative ring-opening metathesis polymerization of homopolymer, PNBCB (35 kDa) is described as follows. In 25 mL round, bottom flask equipped with a magnetic stir bar, monomer NBCB (750 mg, 1.5 mol) was dissolved in 8 mL of DCM and purged with nitrogen for 5 min at room temperature. In a scintillation vial, third generation Grubb’s catalyst (19 mg, 22 mmol) was weighed, dissolved in 1 mL of DCM and added to the stirring solution in the flask. The polymerization was allowed to proceed for 30 min at room temperature under nitrogen atmosphere. The polymerization was then terminated by adding excess ethyl vinyl ether. The resulting polymer was precipitated in methanol, then centrifuged, followed by drying overnight under vacuum at room temperature.

### Synthesis of CdSe NPLs

CdSe NPL was synthesized using modified literature procedures^35^. Cd(myr)_2_ (170 mg, 0.3 mmol), Se (12 mg, 0.15 mmol) and 15 mL of ODE were mixed in a three-neck flask and degassed under vacuum at 100 °C for 1 h. The mixture was heated under nitrogen atmosphere with the temperature set at 200 °C to form Cd–Se complex. When the solution turned orange, it was rapidly cooled down to room temperature and 55 mg of Zn(Ac)_2_ powder was added. The inner air was replaced with nitrogen gas, and then heated up to 240 °C to grow CdSe NPLs. After 10 min of growth, the heating mantle was removed and when the temperature reached 160 °C, 1 mL of OA was injected into the crude solution to enhance the colloidal stability. At room temperature, CdSe NPLs were separated from byproducts such as CdSe sheets and spherical CdSe NPs by centrifuging the product at different × *g*. After centrifugation at 790 × *g* for 10 min, the supernatant containing spherical CdSe NPs was discarded and the precipitant was dispersed in 5 ml of hexane. The solution was centrifuged at 1510 × *g* for 10 min, and colloidal CdSe NPLs were obtained by taking the supernatant. The CdSe NPL solution was purified by precipitation (acetone and ethanol) and redispersion (hexane) method and finally dispersed in chloroform for further composite experiments.

### Synthesis of CdSe/ZnS core/shell NPLs

CdSe/ZnS core/shell NPL was synthesized using modified literature procedures^43^. Zn(Ac)_2_ (73 mg, 0.4 mmol), OA (1 mL), ODE (10 ml) and CdSe NPLs (4.14 × 10^−9^ mol, concentrated in hexane) were introduced into a three-neck flask and degassed under vacuum at 100 °C for 1 h under vigorous stirring. After then, 1 mL of OLA was added and the solution was heated with the temperature set at 300 °C under nitrogen atmosphere. Octanethiol (105 μL) in ODE (6 mL) solution was separately prepared and injected into the seed solution at 170 °C at 10 mL/h, and then at 250 °C, the injection rate was switched to 4 mL/h. When the thiol injection was finished, the shell growth reaction was further allowed for 10 min and then quenched by rapidly cooling the product with cold water. The CdSe/ZnS NPL solution was purified by precipitation (acetone and ethanol) and redispersion (hexane) method and finally dispersed in chloroform for further composite experiments.

### Preparation of NPL/LC nanocomposites

6.7 wt.% of 35k PNBCB and 6OCB solutions were prepared. The mixture solution containing CdSe NPLs, PNBCB and 6OCB (the mass ratio, 0.0086: 1: 1.18) was drop casted on a glass substrate at 120 °C (the mass ratio of CdSe/ZnS NPL, PNBCB and 6OCB is 0.015: 1: 1.18). After the removal of solvent, the isotropic composite was rapidly quenched to room temperature to achieve good NPL dispersions in the smectic phase composite. Then, the sample was scraped away from the substrate and used for further experiments and characterizations. A superconducting magnet (American Magnetics Inc.) was used to provide fields from 0 to 5.8 T. Bulk composite samples were sandwiched between Kapton windows on a temperature-controlled stage for temperature and field-dependent experiments. To make thin composite films for microscope measurements, bulk samples were sandwiched by two glass substrates without spacer.

### Characteristics

^1^H NMR spectroscopy was recorded on Bruker AVANCE 500 MHz NMR spectrometer with CDCl_3_ as solvent. Gel permeation chromatography (GPC) was performed using a Waters 1515 coupled with a PLELS1000 evaporative light scattering detector (ELSD) and a Waters 2487 dual-wavelength absorbance UV–Vis detector with THF as eluent (flow rate 1.0 mL/min) and polystyrene (PS) standards for constructing a conventional calibration curve. NPLs were imaged using field emission transmission electron microscope, JEOL F200. UV–Vis spectra were collected in a Cary 100 bio instrument. Polarized optical microscope imaging was performed using a Zeiss Axio Observer microscope equipped with cross polarizers and a Pike CCD camera. SAXS measurements were conducted on a Rigaku S-MAX3000 instrument using Cu Kα radiation (λ = 1.542 Å) with a pinhole-collimated 1.3 mm diameter beam at the sample plane. The SAXS instrument houses a superconducting magnet and a temperature controller for in situ field and temperature experiments. Thermal analysis data were obtained using a TA DSC 2500 instrument. PL spectra were collected using an Edinburgh instruments FLS 1000 fluorometer with an excitation light of 450 nm. Fluorescence imaging was conducted using a microscope equipped with a fluorescence lamp illuminator, X-cite series 120Q. A fluorescence filter set 43 cube was used to irradiate 545/25 nm light to samples and detect 605/70 nm fluorescence. The emission beam was directed through a polarizer and sent into a CCD camera.

## Supplementary information

Supplementary Information

## Data Availability

All data supporting the findings of this study are available in this article and Supplementary Information File, or are available from the corresponding author upon request.

## References

[1] Bisoyi HK, Kumar S (2011). Liquid-crystal nanoscience: an emerging avenue of soft self-assembly. Chem. Soc. Rev..

[2] Saliba S, Mingotaud C, Kahn ML, Marty J-D (2013). Liquid crystalline thermotropic and lyotropic nanohybrids. Nanoscale.

[3] Bagiński M, Szmurło A, Andruszkiewicz A, Wójcik M, Lewandowski W (2016). Dynamic self-assembly of nanoparticles using thermotropic liquid crystals. Liq. Cryst..

[4] Lee E (2016). Fine golden rings: tunable surface plasmon resonance from assembled nanorods in topological defects of liquid crystals. Adv. Mater..

[5] Mundoor H, Smalyukh II (2015). Mesostructured composite materials with electrically tunable upconverting properties. Small.

[6] Zhang Y, Liu Q, Mundoor H, Yuan Y, Smalyukh II (2015). Metal nanoparticle dispersion, alignment, and assembly in nematic liquid crystals for applications in switchable plasmonic color filters and E-polarizers. ACS Nano.

[7] Coursault D (2012). Linear self-assembly of nanoparticles within liquid crystal defect arrays. Adv. Mater..

[8] Do S-PH (2020). From chains to monolayers: nanoparticle assembly driven by smectic topological defects. Nano Lett..

[9] Milette J (2012). Reversible long-range patterning of gold nanoparticles by smectic liquid crystals. Soft Matter.

[10] Liu Q, Yuan Y, Smalyukh II (2014). Electrically and optically tunable plasmonic guest–host liquid crystals with long-range ordered nanoparticles. Nano Lett..

[11] Du T (2015). Combination of photoinduced alignment and self-assembly to realize polarized emission from ordered semiconductor nanorods. ACS Nano.

[12] Schneider J (2017). Photoinduced micropattern alignment of semiconductor nanorods with polarized emission in a liquid crystal polymer matrix. Nano Lett..

[13] Zhang W (2019). Ligand shell engineering to achieve optimal photoalignment of semiconductor quantum rods for liquid crystal displays. Adv. Funct. Mater..

[14] Kumar S, Bisoyi HK (2007). Aligned carbon nanotubes in the supramolecular order of discotic liquid crystals. Angew. Chem. Int. Ed..

[15] Ji Y, Huang YY, Rungsawang R, Terentjev EM (2010). Dispersion and alignment of carbon nanotubes in liquid crystalline polymers and elastomers. Adv. Mater..

[16] Rožič B (2017). Oriented gold nanorods and gold nanorod chains within smectic liquid crystal topological defects. ACS Nano.

[17] Lee J-S, Lee B, Song S-H, Song J-K (2019). Biphasic dielectrophoresis of isotropic pocket carriers containing quantum dots (QDs) in nematic medium and fabrication of QD cluster array with matrix emission of point light sources. Part. Part. Syst. Charact..

[18] Riahinasab ST (2019). Nanoparticle-based hollow microstructures formed by two-stage nematic nucleation and phase separation. Nat. Commun..

[19] Milette J (2012). Reversible long range network formation in gold nanoparticle—nematic liquid crystal composites. Soft Matter.

[20] Dudka T (2019). Formulation of a composite system of liquid crystals and light-emitting semiconductor quantum rods: from assemblies in solution to photoaligned films. Adv. Mater. Technol..

[21] Cseh L, Mehl GH (2006). The design and investigation of room temperature thermotropic nematic gold nanoparticles. J. Am. Chem. Soc..

[22] Saliba S (2011). Liquid crystal based on hybrid zinc oxide nanoparticles. J. Mater. Chem..

[23] Roohnikan M (2018). Mechanochemical nanoparticle functionalization for liquid crystal nanocomposites based on COOH-pyridine heterosynthons. J. Mater. Chem. C.

[24] Milette J, Toader V, Reven L, Lennox RB (2011). Tuning the miscibility of gold nanoparticles dispersed in liquid crystals via the thiol-for-DMAP reaction. J. Mater. Chem..

[25] Qi H, Hegmann T (2009). Multiple alignment modes for nematic liquid crystals doped with alkylthiol-capped gold nanoparticles. ACS Appl. Mater. Interfaces.

[26] Yu J, Chen R (2020). Optical properties and applications of two-dimensional CdSe nanoplatelets. InfoMat.

[27] Rossinelli AA (2019). Compositional grading for efficient and narrowband emission in cdse-based core/shell nanoplatelets. Chem. Mater..

[28] Abécassis B, Tessier MD, Davidson P, Dubertret B (2014). Self-assembly of cdse nanoplatelets into giant micrometer-scale needles emitting polarized light. Nano Lett..

[29] Cunningham PD (2016). Assessment of anisotropic semiconductor nanorod and nanoplatelet heterostructures with polarized emission for liquid crystal display technology. ACS Nano.

[30] Momper R (2020). Kinetic control over self-assembly of semiconductor nanoplatelets. Nano Lett..

[31] Mertelj A, Lisjak D, Drofenik M, Copic M (2013). Ferromagnetism in suspensions of magnetic platelets in liquid crystal. Nature.

[32] Gabinet UR (2021). Nanocomposites of 2D-MoS2 exfoliated in thermotropic liquid crystals. ACS Mater. Lett..

[33] Glotzer SC (2012). Shape matters. Nature.

[34] Gopinadhan M (2017). Controlling orientational order in block copolymers using low-intensity magnetic fields. Proc. Natl Acad. Sci..

[35] Ithurria S, Bousquet G, Dubertret B (2011). Continuous transition from 3D to 1D confinement observed during the formation of CdSe nanoplatelets. J. Am. Chem. Soc..

[36] González García Á, Nagelkerke MMB, Tuinier R, Vis M (2020). Polymer-mediated colloidal stability: on the transition between adsorption and depletion. Adv. Colloid Interface Sci..

[37] Kim S, Hyun K, Moon JY, Clasen C, Ahn KH (2015). Depletion stabilization in nanoparticle–polymer suspensions: multi-length-scale analysis of microstructure. Langmuir.

[38] Zhang X, Servos MR, Liu J (2012). Ultrahigh nanoparticle stability against salt, pH, and solvent with retained surface accessibility via depletion stabilization. J. Am. Chem. Soc..

[39] Semenov AN (2008). Theory of colloid stabilization in semidilute polymer solutions. Macromolecules.

[40] Shuai M (2016). Spontaneous liquid crystal and ferromagnetic ordering of colloidal magnetic nanoplates. Nat. Commun..

[41] Mertelj A (2019). Evolution of nematic and ferromagnetic ordering in suspensions of magnetic nanoplatelets. Soft Matter.

[42] Mahler B, Nadal B, Bouet C, Patriarche G, Dubertret B (2012). Core/shell colloidal semiconductor nanoplatelets. J. Am. Chem. Soc..

[43] Altintas Y (2019). Highly stable, near-unity efficiency atomically flat semiconductor nanocrystals of CdSe/ZnS hetero-nanoplatelets enabled by ZnS-shell hot-injection growth. Small.

[44] Van der Beek D (2006). Magnetic-field-induced orientational order in the isotropic phase of hard colloidal platelets. Phys. Rev. E.

[45] Davidson P, Penisson C, Constantin D, Gabriel J-CP (2018). Isotropic, nematic, and lamellar phases in colloidal suspensions of nanosheets. Proc. Natl Acad. Sci..

[46] Eliseev AA (2018). Rotational dynamics of colloidal hexaferrite nanoplates. Appl. Phys. Lett..

[47] Beaudoin E, Abecassis B, Constantin D, Degrouard J, Davidson P (2015). Strain-controlled fluorescence polarization in a CdSe nanoplatelet–block copolymer composite. Chem. Commun..

[48] Deshmukh P (2014). Molecular design of liquid crystalline brush-like block copolymers for magnetic field directed self-assembly: a platform for functional materials. ACS Macro Lett..

[49] Ndaya D, Bosire R, Mahajan L, Huh S, Kasi R (2018). Synthesis of ordered, functional, robust nanoporous membranes from liquid crystalline brush-like triblock copolymers. Polym. Chem..

