# Supplementary material for: Dynamic magnetic field alignment and polarized emission of semiconductor nanoplatelets in a liquid crystal polymer
Source: Nat Commun. 2022 May 6;13:2507. doi: 10.1038/s41467-022-30200-2 (PMC9076605; doi:10.1038/s41467-022-30200-2)
Supplement: Supplementary file 1 — Supplementary Information [file 41467_2022_30200_MOESM1_ESM.pdf]

Supplementary Information

Dynamic Magnetic Field Alignment and Polarized Emission of Semiconductor Nanoplatelets in a Liquid Crystal Polymer

Dahin Kim,[a] Dennis Ndaya,[b,c] Reuben Bosire,[b] Francis K. Masese,[b] Weixingyue Li,[d] Sarah M. Thompson,[e] Cherie R. Kagan,[d,e,f] Christopher B. Murray,[d,f] Rajeswari M. Kasi,[b,c] Chinedum O. Osuji[*,a]*

[a]Department of Chemical and Biomolecular Engineering, University of Pennsylvania, Philadelphia, Pennsylvania 19104, United States

[b]Department of Chemistry, University of Connecticut, Storrs, Connecticut 06269, United States

[c]Polymer Program, Institute of Materials Science, University of Connecticut, Storrs, Connecticut 06269, United States

[d]Department of Chemistry, University of Pennsylvania, Philadelphia, Pennsylvania 19104, United States

[e]Department of Electrical and Systems Engineering, University of Pennsylvania, Philadelphia, Pennsylvania 19104, United States

[f]Department of Materials Science and Engineering, University of Pennsylvania, Philadelphia, Pennsylvania 19104, United States

Inventory of Supporting Information

Supplementary Figure 1-22

- **Fig 1:** UV-Vis, emission and SAXS data of neat NPLs
- **Fig 2:** ^{1}H NMR data of LC polymer
- **Fig 3:** DSC data of LCs and NPL-LCs
- **Fig 4:** POM data of LCs
- **Fig 5:** Dispersion stability of NPLs in solution in the presence of LCs
- **Fig 6:** Dispersion stability of NPs in solution in the presence of LCs
- **Fig 7:** Effect of LC polymer molecular weight on NPL dispersion stability
- **Fig 8:** T-dependent SAXS data of LCs under magnetic fields
- **Fig 9:** T-dependent POM data of LCs during heating
- **Fig 10:** T-dependent POM data of LCs during cooling
- **Fig 11:** POM data of magnetically aligned LCs
- **Fig 12:** POM data of LCs at different cooling rates
- **Fig 13:** DSC data of LCs and NPL-LCs at different heating and cooling rates
- **Fig 14:** T-dependent SAXS data of NPL-LCs under magnetic fields
- **Fig 15:** Correlation length of NPLs determined by SAXS and TEM
- **Fig 16:** TEM data of magnetically aligned NPL-LCs
- **Fig 17:** SAXS data of neat core/shell NPLs
- **Fig 18:** Dispersion stability of core/shell NPLs in solution in the presence of LCs
- **Fig 19:** POM and FM data of core/shell NPL-LCs cooled at 1 °C/min
- **Fig 20:** FM data of core/shell NPL-LCs at the smectic and nematic phases
- **Fig 21:** TEM data of core/shell NPL-LCs
- **Fig 22:** SAXS data of core/shell NPL-LCs under magnetic fields

Reference

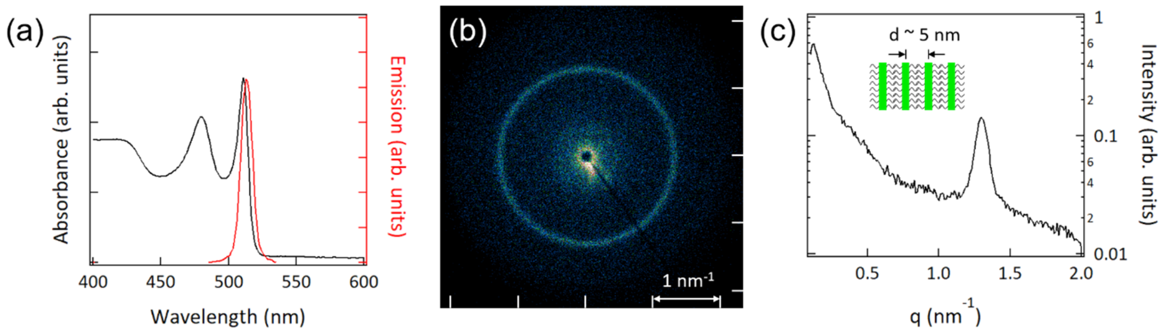

Supplementary Fig 1: UV-Vis, emission and small angle X-ray scattering (SAXS) data of neat nanoplatelets (NPLs). a UV-Vis and emission spectra of CdSe NPLs. **b** SAXS pattern of drop-casted CdSe NPLs. **c** SAXS intensity vs. scattering vector q for the pattern of **b**. Inset picture demonstrates the d-spacing between closely stacked NPLs with the consideration of NPL thickness and slightly interdigitated ligand layers.

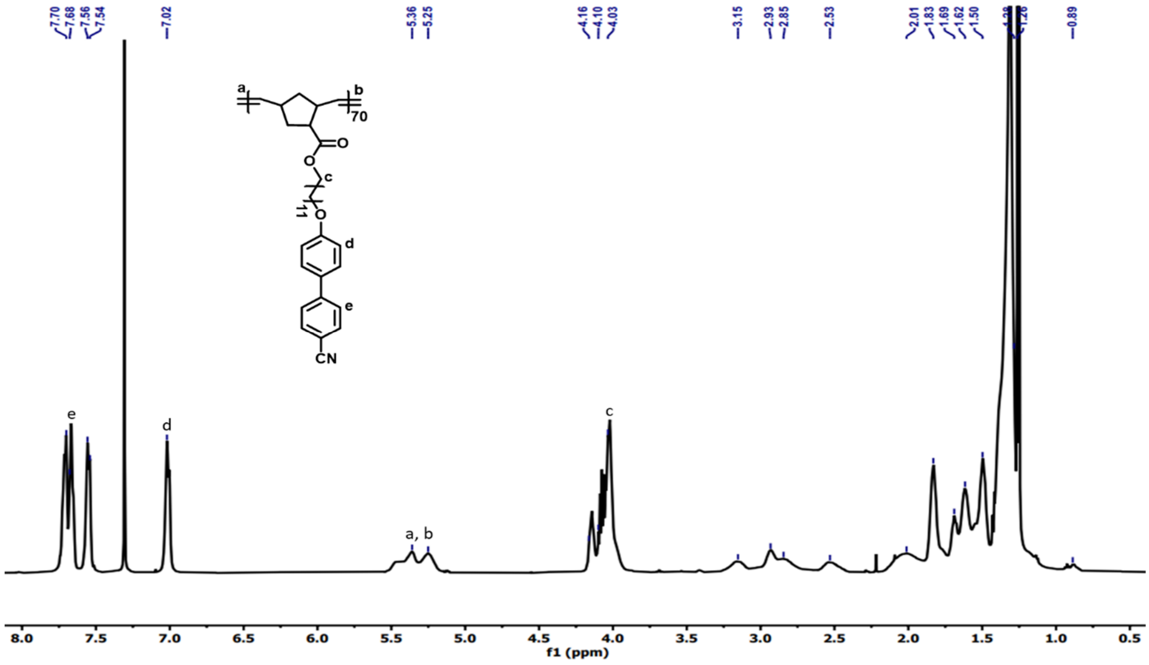

Supplementary Fig 2: ^{1}H NMR data of liquid crystal (LC) polymer. ^{1}H NMR for the PNBCB in $CDCl_3$ at room temperature (theoretical molecular weight = 35 kDa, GPC = 34.3 kDa (Đ = 1.08)).

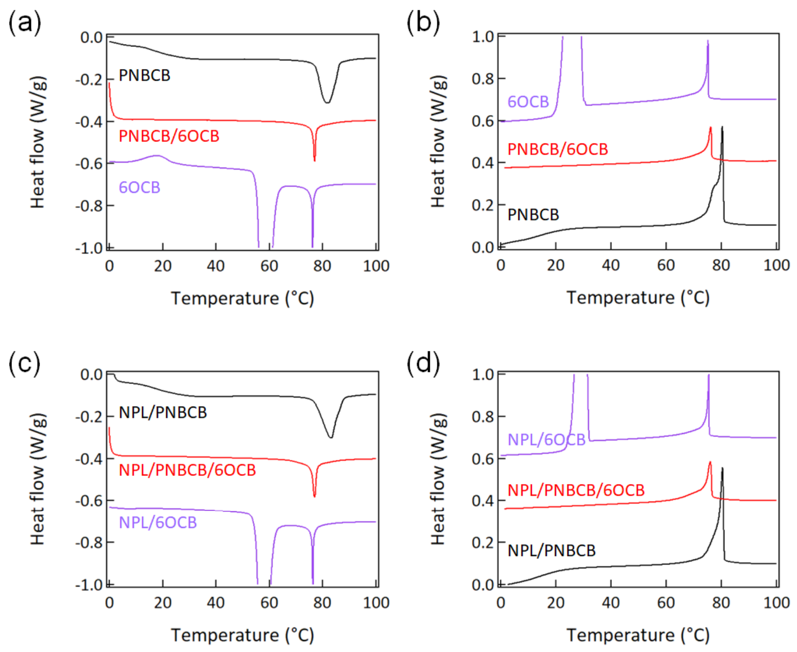

(e)

	In heating cycle				In cooling cycle			
	$T_{K\text{-}N}$ (°C)	$\Delta H_{K\text{-}N}$ (J g^{-1})	$T_{N\text{-}Iso}$ (°C)	$\Delta H_{N\text{-}Iso}$ (J g^{-1})	$T_{K\text{-}N}$ (°C)	$\Delta H_{K\text{-}N}$ (J g^{-1})	$T_{N\text{-}Iso}$ (°C)	$\Delta H_{N\text{-}Iso}$ (J g^{-1})
PNBCB			83.0	6.97			80.4	7.12
PNBCB/6OCB			76.4	1.93			76.2	2.60
6OCB	56.8	89.7	75.8	1.27	32.5	66.1	75.9	1.47
NPL/PNBCB			82.7	6.51			80.3	7.03
NPL/PNBCB/6OCB			76.9	1.42			76.0	2.02
NPL/6OCB	57.7	88.1	76.2	1.70	26.6	65.1	75.2	1.80

Supplementary Fig 3: **Differential scanning calorimetry (DSC) data of LCs and NPL-LCs. a, b** DSC curves obtained during **a** heating and **b** cooling of PNBCB, PNBCB/6OCB and 6OCB. **c, d** DSC traces in **c** heating and **d** cooling cycles when NPLs are added to each LC system. **e** Transition temperatures and enthalpy changes obtained from the DSC results in **a-d**. The DSC curve of PNBCB/6OCB shows that the clearing point is 76.4 °C and there is no crystallization peak in the measurement range between 0 °C and 100 °C, implying a successful mixing of PNBCB and 6OCB.

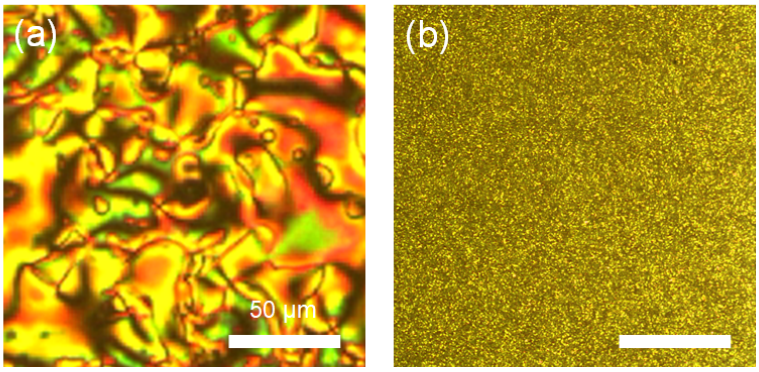

Supplementary Fig 4: Polarized optical microscopy (POM) data of LCs. a, b POM images of **a** 6OCB and **b** PNBCB at 70 °C.

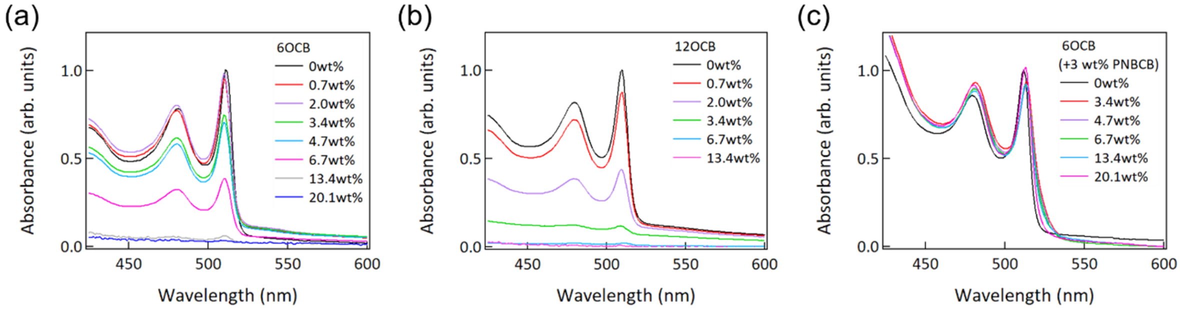

Supplementary Fig 5: **Dispersion stability of NPLs in solution in the presence of LCs. a, b** Absorption spectra of supernatants after gentle centrifugation of **a** NPL/6OCB and **b** NPL/12OCB solutions with varying the concentration of 6OCB and 12OCB, respectively. The wavelength range from 425 nm to 600 nm displays the absorption by CdSe NPLs, implying that more NPLs precipitate out with increasing the small molecule LC contents. **c** Absorption spectra of supernatants of NPL/6OCB solutions in the presence of 3 wt.% of PNBCB.

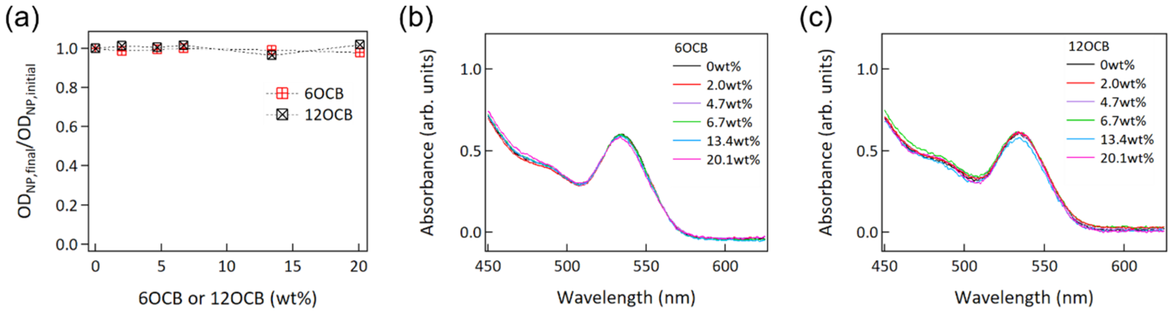

Supplementary Fig 6: **Dispersion stability of nanoparticles (NPs) in solution in the presence of LCs. a** Dispersion stability of 2.7 nm CdSe NPs when co-dispersed with 6OCB or 12OCB, which is evaluated by the decrease in optical density (OD) of supernatants after gentle centrifugation of the solutions. **b, c** Absorption spectra of supernatants of **b** NP/6OCB and **c** NP/12OCB solutions depending on the concentration of 6OCB and 12OCB, respectively. A constant optical density at the given wavelength range shows that CdSe NPs are stably dispersed regardless of the 6OCB and 12OCB concentrations.

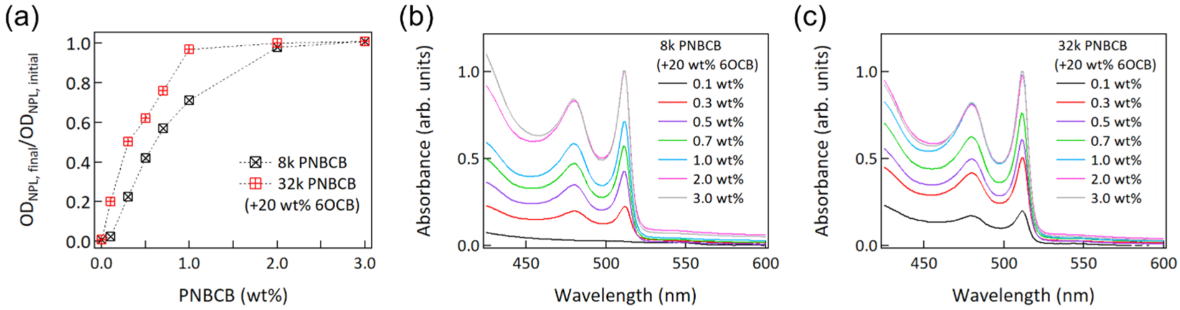

Supplementary Fig 7: **Effect of LC polymer molecular weight on NPL dispersion stability. a** Dispersion stability of CdSe NPLs in NPL/PNBCB/6OCB solutions as a function of PNBCB wt.% when the molecular weight of PNBCB is 8k and 32k, respectively. **b, c** Absorption spectra of supernatants after centrifugation of NPL/PNBCB/6OCB solutions with varying the PNBCB concentration for **b** 8k and **c** 32k PNBCB.

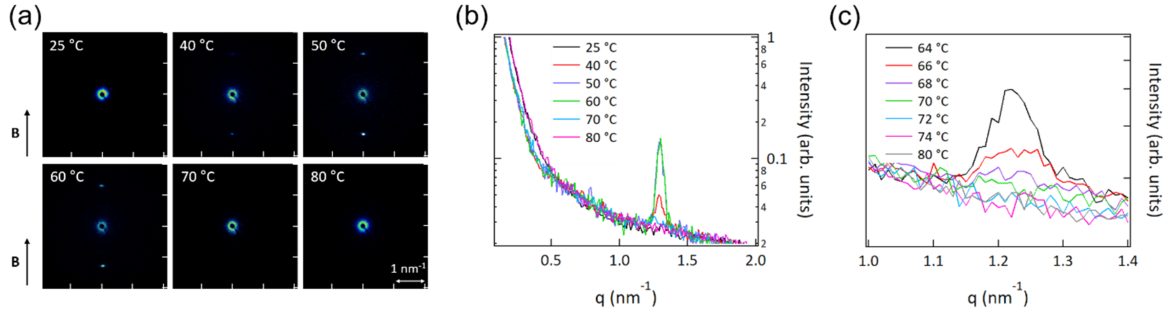

Supplementary Fig 8: Temperature-dependent SAXS data of LCs under magnetic fields. a SAXS patterns of PNBCB/6OCB LC blends depending on temperature under vertical magnetic fields (**B**) of 5.8 T with X-rays incident perpendicular to the applied field. **b** SAXS intensity vs. scattering vector q, extracted from the patterns of **a**. The spot-like reflections at q = 1.3 nm^{-1} along the meridional line in the patterns indicate that the smectic layers are formed aligned perpendicular to the field. **c** SAXS intensity vs. q between 64 °C and 80 °C. The intensity of the smectic layer reflection gradually decreases with temperature and disappears at ~72 °C, suggesting that the smectic phase is lost at this temperature. The transition is to a nematic phase as also inferred from POM data.

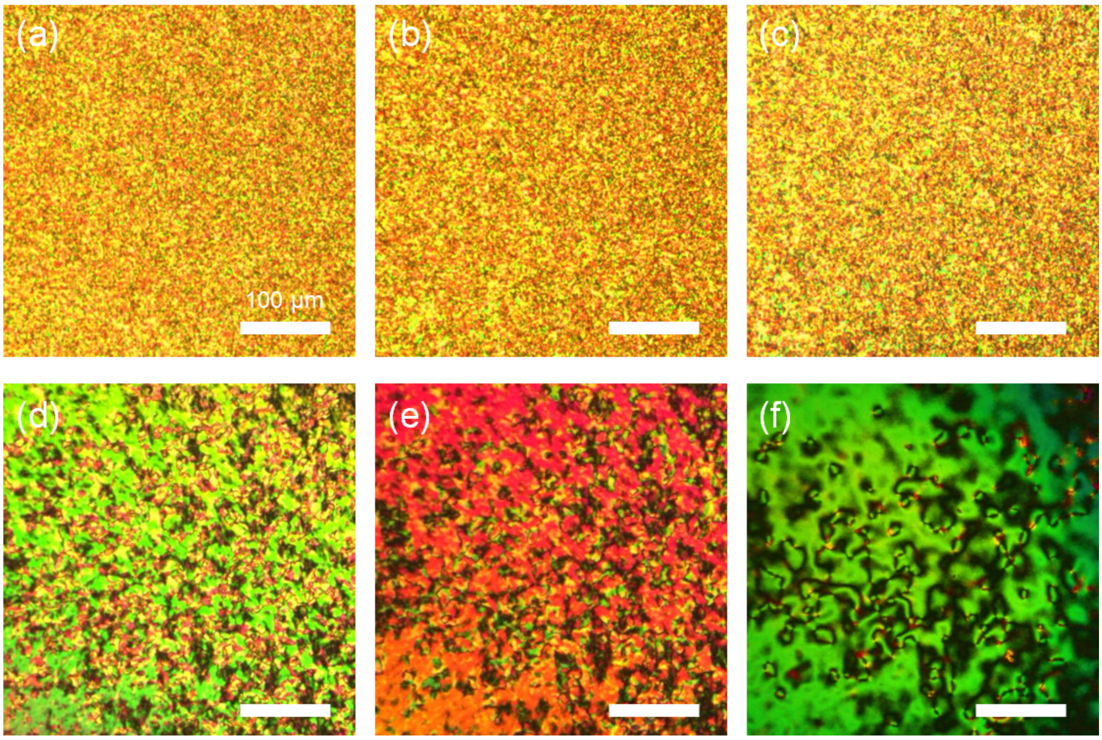

Supplementary Fig 9: Temperature-dependent POM data of LCs during heating. a-f POM images of PNBCB/6OCB measured at various temperatures during heating from room temperature to the isotropic state; **a** 30 °C, **b** 69.5 °C, **c** 71.0 °C, **d** 72.8 °C, **e** 77.3 °C and **f** 78.5 °C. PNBCB/6OCB obtained by quenching the melt from the isotropic phase to room temperature shows a fine-grainy birefringence. Upon heating, the grains start to grow at ~72 °C and generate schlieren texture before the clearing.

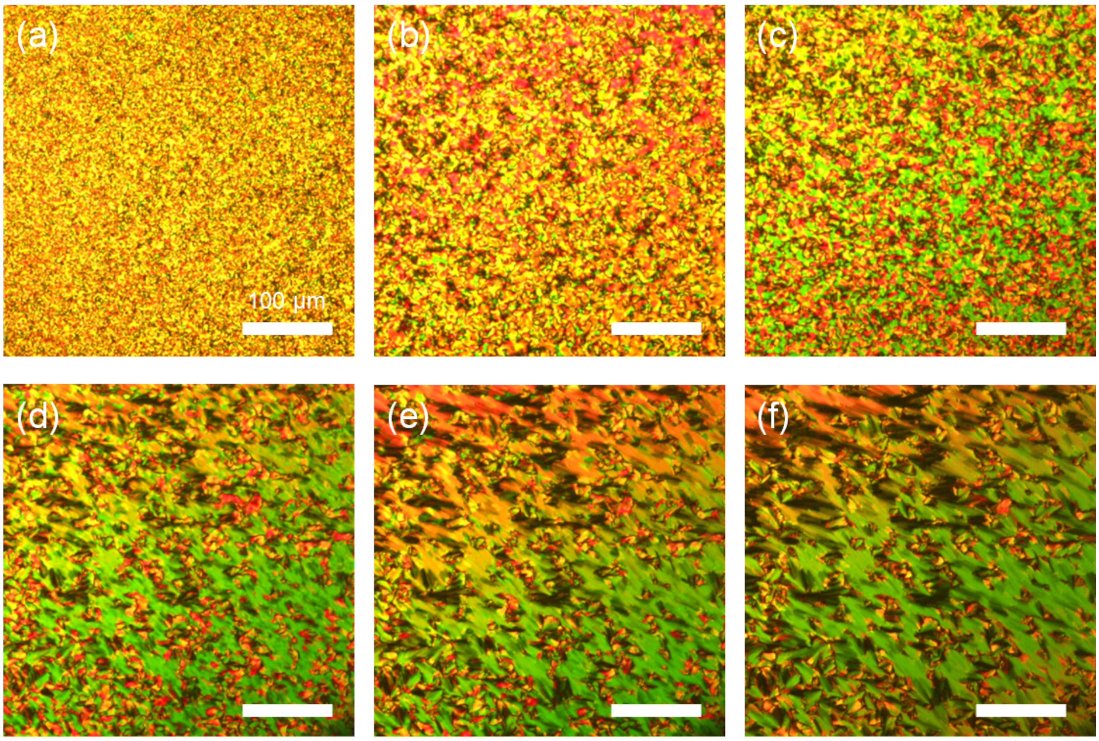

Supplementary Fig 10: Temperature-dependent POM data of LCs during cooling. a-f POM images of PNBCB/6OCB measured at various temperatures during cooling from the isotropic state to room temperature at 1 °C/min; **a** 78.8 °C, **b** 77.2 °C, **c** 72.7 °C, **d** 71.1 °C, **e** 69.6 °C and **f** 30 °C.

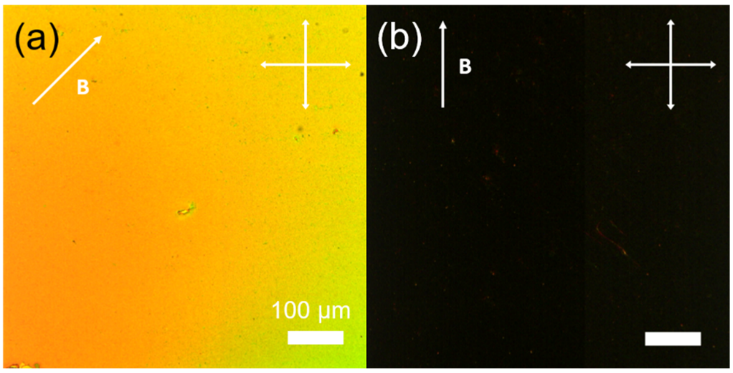

Supplementary Fig 11: POM data of magnetically aligned LCs. a, b POM images of PNBCB/6OCB after magnetic field alignment; **a** when the field direction is at 45° to polarizer axes and **b** parallel to one of the polarizer axes.

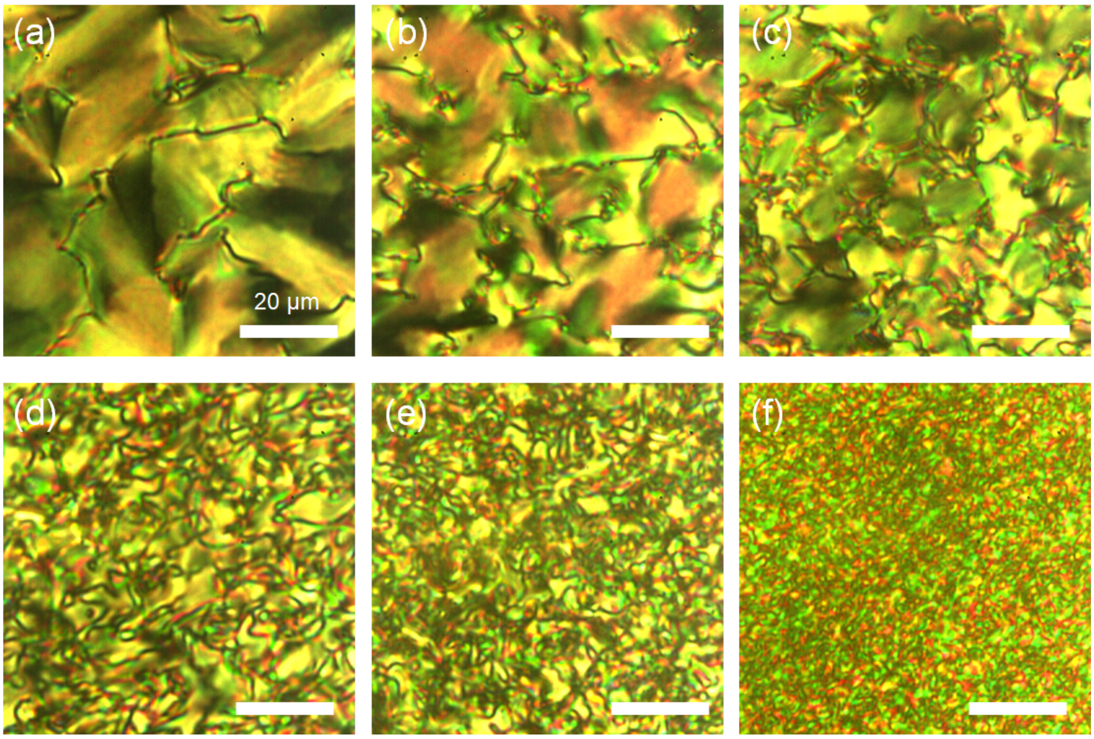

Supplementary Fig 12: **POM data of LCs at different cooling rates. a-f** POM images of PNBCB/6OCB cooled from the isotropic phase to room temperature at various rates; **a** 1 °C/min, **b** 5 °C/min, **c** 10 °C/min, **d** 20 °C/min, **e** 30 °C/min, and **f** quenching (> 30 °C/min).

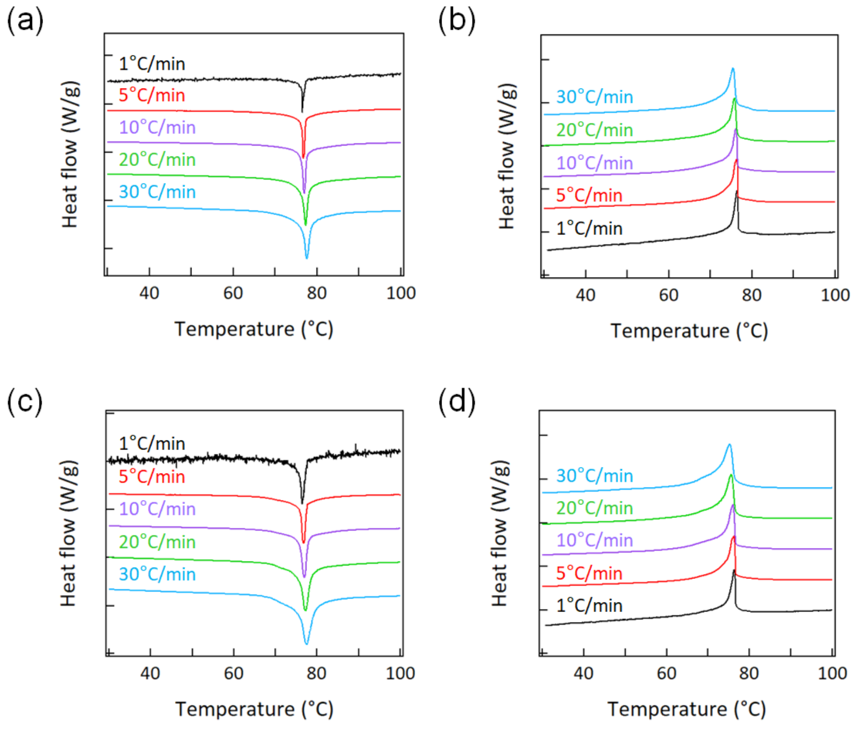

(e)

		In heating cycle		In cooling cycle	
		$T_{N\text{-}Iso}$ (°C)	$\Delta H_{N\text{-}Iso}$ (J g^{-1})	$T_{N\text{-}Iso}$ (°C)	$\Delta H_{N\text{-}Iso}$ (J g^{-1})
PNBCB/ 6OCB	1 °C/min	76.5	1.71	76.4	1.61
	5 °C/min	76.5	1.68	76.4	2.13
	10 °C/min	76.4	1.93	76.2	2.60
	20 °C/min	76.4	1.84	76.5	2.22
	30 °C/min	77.6	1.64	76.3	2.22
PNBCB/ 6OCB/ NPL	1 °C/min	75.8	1.77	76.4	1.88
	5 °C/min	76.2	1.49	76.3	2.40
	10 °C/min	76.9	1.42	76.0	2.02
	20 °C/min	77.2	2.29	75.6	2.67
	30 °C/min	77.5	2.55	75.2	2.15

Supplementary Fig 13: DSC data of LCs and NPL-LCs at different heating and cooling rates. a, b DSC curves obtained during **a** heating and **b** cooling of PNBCB/6OCB at different rates. **c, d** DSC traces in **c** heating and **d** cooling cycles when NPLs are added to PNBCB/6OCB. **e** Transition temperatures and enthalpy changes obtained from the DSC results shown in **a-d**.

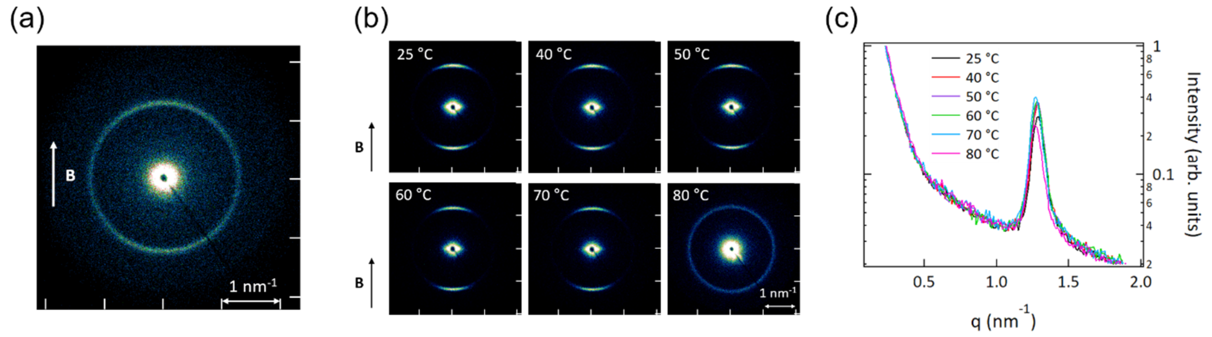

Supplementary Fig 14: **Temperature-dependent SAXS data of NPL-LCs under magnetic fields. a** SAXS pattern of NPL/PNBCB/6OCB composite which was held at 74 °C for 30 min. **b** SAXS patterns of NPL/PNBCB/6OCB composite depending on temperature under vertical magnetic fields (**B**) of 5.8 T. **c** SAXS intensity vs. scattering vector q, extracted from the patterns in **b**.

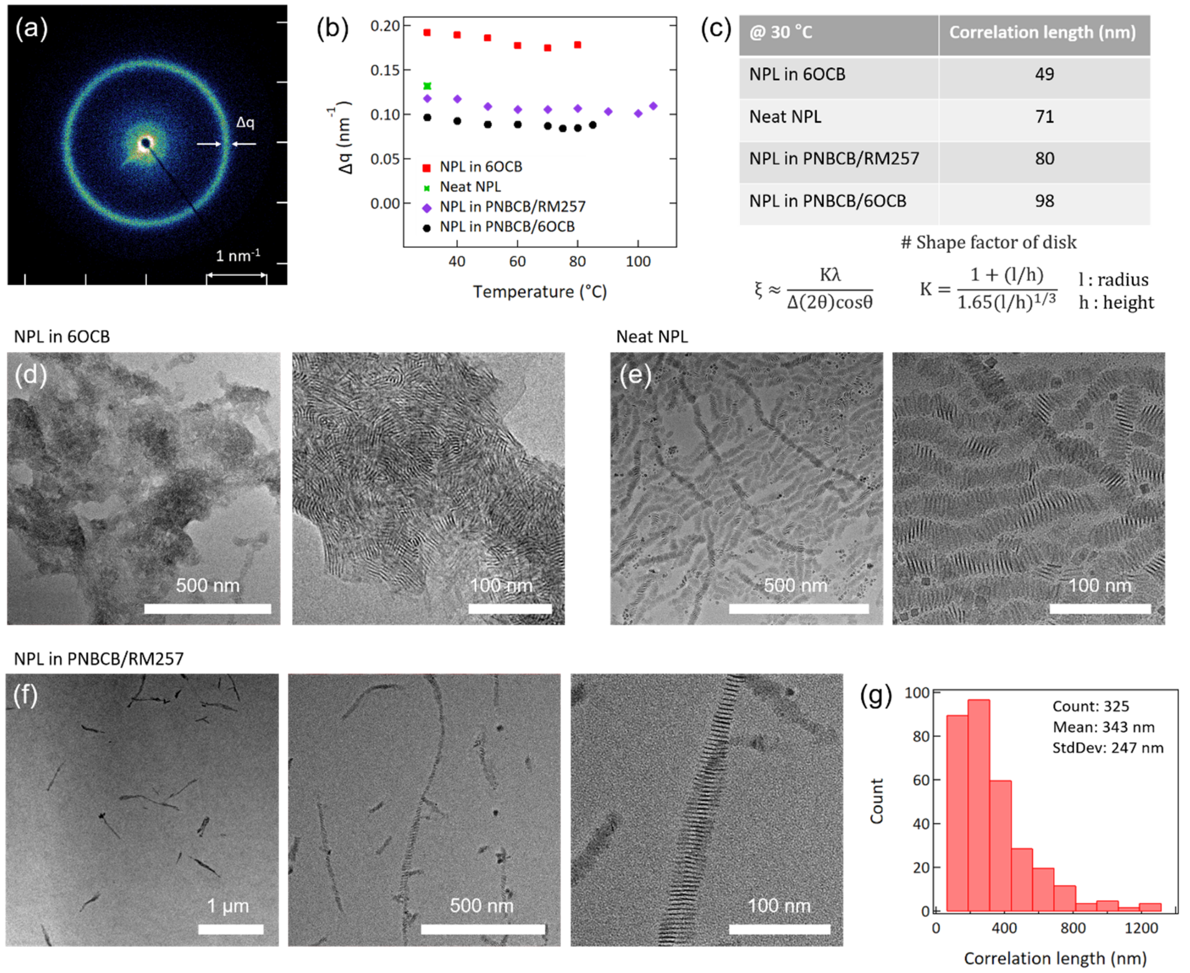

Supplementary Fig 15: Correlation length of NPLs determined by SAXS and transmission electron microscopy (TEM). a SAXS pattern of neat NPLs. The full width half maximum (Δq) for the X-ray reflection characterizes the grain size. **b** Δq of the SAXS peak for neat NPLs and NPLs dispersed in 6OCB, PNBCB/6OCB and PNBCB/RM257 as a function of temperature, as determined from fits to the SAXS patterns. **c** Correlation length of NPL stacks depending on the dispersion medium based on the Scherrer equation, which is underestimated because there is a lower limit due to instrumental broadening. The shape factor, *K*, was estimated as 1.5.[1] **d** TEM images of NPLs in 6OCB, showing randomly gathered NPLs as they get destabilized by 6OCB and rapidly aggregated into big chunks. The TEM sample was prepared via drop-casting of the solution. **e** TEM images of close-packed neat NPLs deposited via drop-casting of the solution, where they form stacks due to the strong face-to-face attraction. **f** TEM images of NPLs in PNBCB/RM257 film. The sample was cross-linked at 100 °C and microtomed. **g** Statistics of the

actual correlation lengths of NPLs in PNBCB/RM257 on the basis of TEM measurements.

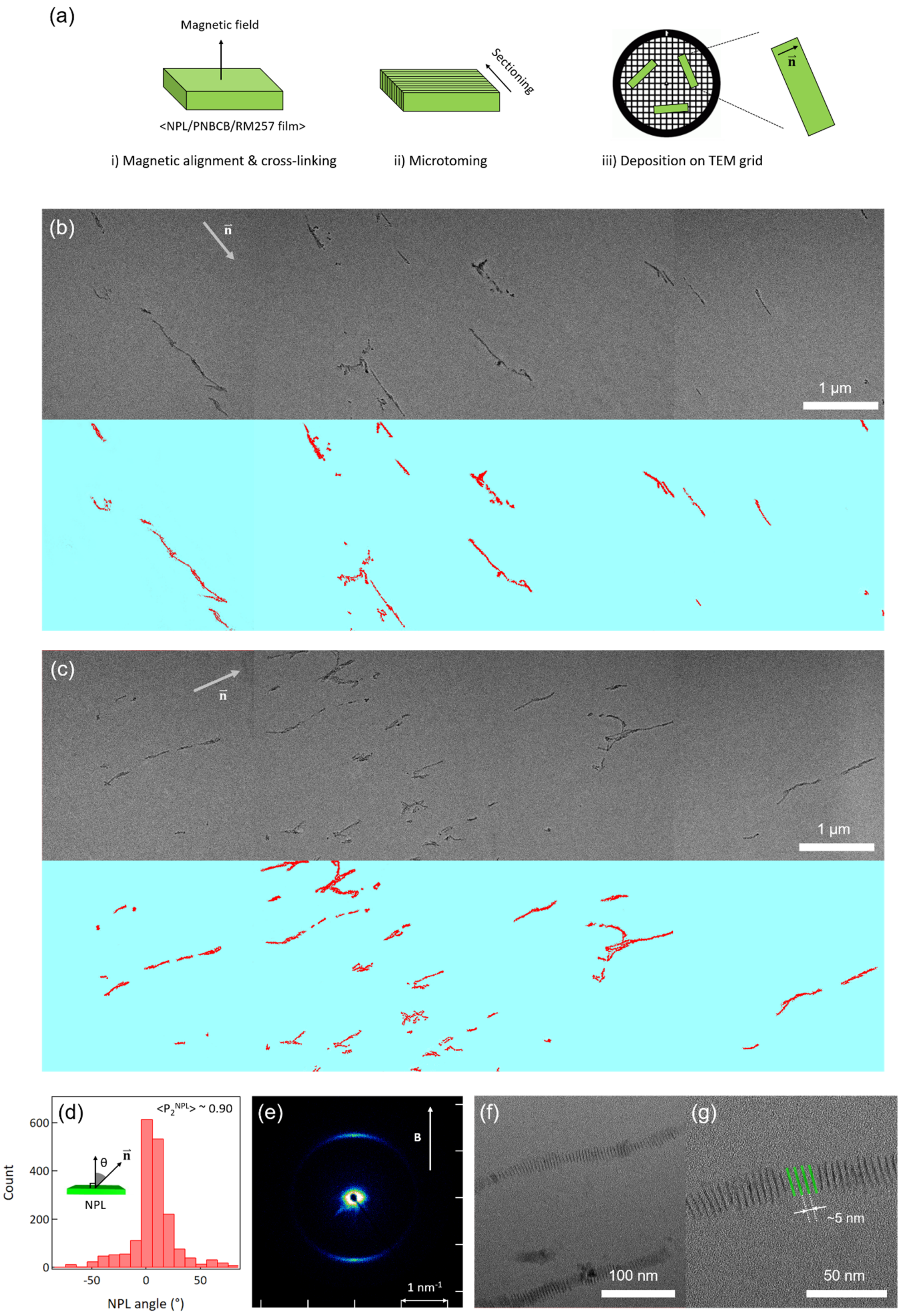

(a)
Magnetic field
<NPL/PNBCB/RM257 film>
Sectioning
i) Magnetic alignment & cross-linking
ii) Microtoming
iii) Deposition on TEM grid
(b)
1 μm
(c)
1 μm
(d)
<P2NPL> ~ 0.90
Count
NPL
NPL angle (°)
(e)
B
1 nm-1
(f)
100 nm
(g)
~5 nm
50 nm

Supplementary Fig 16: TEM data of magnetically aligned NPL-LCs. a TEM sample preparation procedure. **b, c** TEM images of aligned NPL/PNBCB/RM257 composite polymer, taken from two different sectioned films on a TEM grid. The $\vec{n}$ denotes the director of LCs. The TEM images are false colored to highlight the alignment of NPL stacks. **d** Statistics of the angle between the NPL surface normal and the LC director. **e** SAXS pattern for aligned NPL/PNBCB/RM257 composite at an external magnetic field (**B**) of 5.8 T. The SAXS result shows a well-defined peak at scattering vector q = 1.26 nm^{-1}. **f** TEM image showing that long agglomerates embedded in LCs are constituted by face-to-face stacked NPLs. **g** High-magnification TEM image indicating that NPL stacks have a center-to-center distance of 5.0 nm, which corresponds to $d = 2\pi/q$ = 5.0 nm obtained from the SAXS pattern.

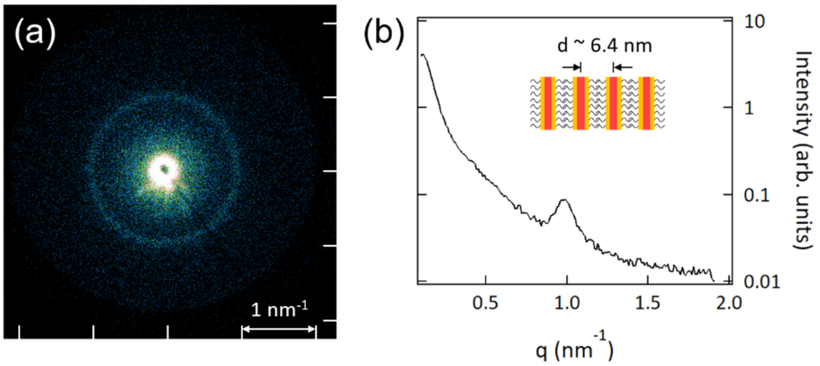

Supplementary Fig 17: SAXS data of neat core/shell NPLs. a SAXS pattern of drop-casted CdSe/ZnS core/shell NPLs. **b** SAXS intensity vs. scattering vector q for the pattern of **a**. Inset picture demonstrates the d-spacing between closely stacked CdSe/ZnS NPLs with the consideration of NPL thickness and slightly interdigitated ligand layers.

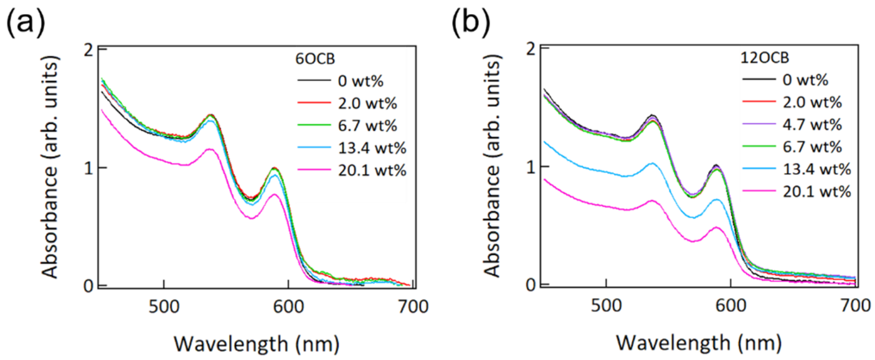

Supplementary Fig 18: Dispersion stability of core/shell NPLs in solution in the presence of LCs. a, b Absorption spectra of CdSe/ZnS NPL supernatants after gentle centrifugation of **a** NPL/6OCB and **b** NPL/12OCB mixture solutions with varying the LC concentration.

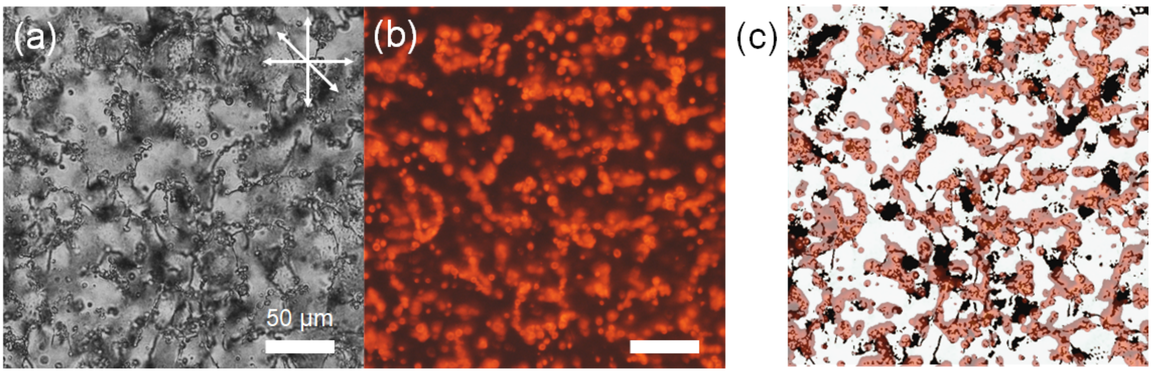

Supplementary Fig 19: POM and fluorescence microscopy (FM) data of core/shell NPL-LCs cooled at 1 °C/min. a POM image of NPL/PNBCB/6OCB composite cooled at 1 °C/min from the isotropic state to room temperature and **b** the corresponding FM image. **c** Overlay of the POM image **a** and the FM image **b** after some adjustments.

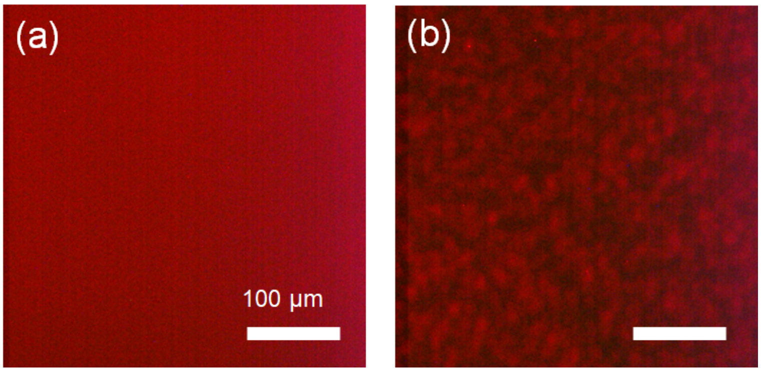

Supplementary Fig 20: FM data of core/shell NPL-LCs at the smectic and nematic phases. **a, b** FM images of NPL/PNBCB/6OCB composites kept **a** at 67 °C and **b** at 73 °C for 1 h.

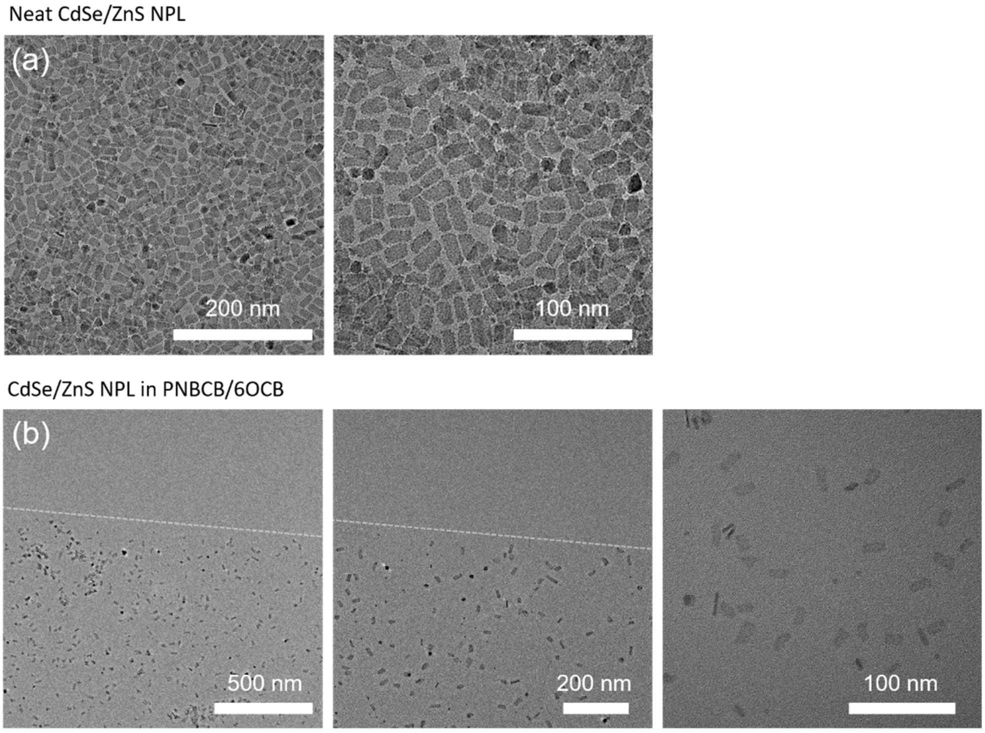

Supplementary Fig 21: TEM data of core/shell NPL-LCs. **a** TEM images of close-packed neat CdSe/ZnS core/shell NPLs. **b** TEM images of CdSe/ZnS NPLs singly dispersed in PNBCB/6OCB matrix. Dashed lines indicate the boundary of the sectioned film.

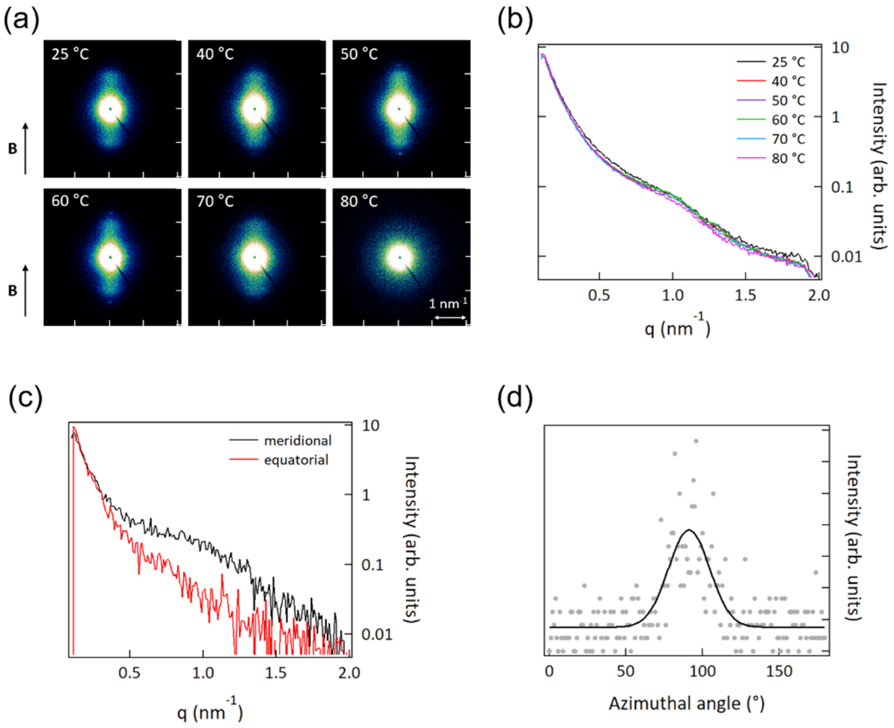

Supplementary Fig 22: **SAXS data of core/shell NPL-LCs under magnetic fields. a** 2D SAXS patterns of PNBCB/6OCB composite containing CdSe/ZnS NPLs depending on temperature under a vertical magnetic field (**B**) of 5.8 T. **b** SAXS intensity traces vs. scattering vector q, extracted from the SAXS patterns in **a**. **c** Meridional and equatorial SAXS intensity traces obtained from the SAXS pattern at 50 °C in **a**. The broad peak indicates a positional short-range order in the nematic phase NPL distribution which can then be regarded as a columnar nematic. **d** Orientation distribution of NPLs which is estimated by fitting an azimuthal scan of the scattered intensity going through the q = 0.098 Å^{-1}.

Reference

1. W. H. Qi, P. Wang, Q. H. Liu, Shape factor of nonspherical nanoparticles, J. Mater. Sci. **40**, 2737-2739 (2005).
